# Evaluating the sample size requirements of tree-based ensemble machine learning techniques for clinical risk prediction

**DOI:** 10.1177/09622802251338983

**Published:** 2025-05-14

**Authors:** Oya Kalaycıoğlu, Menelaos Pavlou, Serhat E Akhanlı, Mark A de Belder, Gareth Ambler, Rumana Z Omar

**Affiliations:** 1 Department of Biostatistics and Medical Informatics, 52942 Bolu Abant İzzet Baysal University, Bolu, Türkiye; 2 Department of Statistical Science, 4919 University College London, London, UK; 3 Department of Statistics, 52986 Muğla Sıtkı Koçman University, Muğla, Türkiye; 4 National Institute for Cardiovascular Outcomes Research, NHS Arden and Greater East Midlands Commissioning Support Unit, Leicester, UK

**Keywords:** Machine learning, sample size, bagging, random forests, boosting, prediction model

## Abstract

Machine learning techniques (MLTs) are increasingly being used to develop clinical risk prediction models for binary health outcomes but the sample size requirements for developing and validating such models remain unclear. This study investigates whether sample size guidelines that target mean absolute prediction error (MAPE) for logistic regression models can be applied to tree-based ensemble MLTs (bagging, random forests, and boosting). Simulations based on two large cardiovascular datasets were used to evaluate the performance of MLTs in terms of MAPE, calibration, the *C*-statistic and Brier score, across six data-generating mechanisms (DGMs) and varying sample sizes. When the DGM and analysis model matched, boosting required a sample size 2–3 times larger than recommended; random forests and bagging did not achieve the target MAPE even with a 12-fold increase. For a neutral DGM that did not match any of the analysis models, logistic regression with only main effects and boosting resulted in target MAPE values with a 12-fold increase in the recommended sample size. For external validation, our simulations showed that sample size guidelines to achieve a target precision of the estimated *C*-statistic were suitable, and thus may be used to inform sample size calculations for MLTs.

## 1 Introduction

Clinical risk prediction models can support medical decision-making by estimating the risk of a health outcome such as disease progression, complications, and mortality. Logistic regression is commonly used to predict the risk for binary outcomes using patient-related information such as demographic and clinical characteristics (predictors). Over the past decade, the use of machine learning techniques (MLTs) has gained increasing attention in risk prediction for health outcomes.^1^ For example, gradient boosting has been used to predict the risk of death in patients with coronavirus disease 2019 (COVID-19),^2^ random forests (RFs) have been applied to predict Type 2 diabetes risk using large-scale health check-up data,^3^ and neural networks have been used to improve pre-existing cardiovascular risk prediction models.^4^

The development of clinical risk prediction models involves estimating the relationship between the outcome and predictors. In regression-based models this entails estimating the regression coefficients, which can then be used to calculate the probability of the event given specific values of the predictor variables. The performance of the developed model is then assessed through either internal validation using a portion of the development data not used in model development or external validation using entirely new data. In MLTs, the model development step involves building a model to identify patterns in the data and making accurate predictions based on these patterns. Previous research suggests that the benefits of MLTs over conventional logistic regression in clinical prediction modelling arise when complex relationships exist between the outcome and predictors such as non-linearities and interactions.^5^ Therefore, MLTs may perform better than regression-based approaches in high-dimensional data with complex structures such as electronic health record data.^6^ We note that, complex data structures involving non-linear and interaction effects can be incorporated in regression models by including additional terms in the regression equation. However, fitting such models require larger sample sizes compared to regression models with only (linear) main effects due to the increased number of parameters.

Several systematic reviews have found that studies using MLTs for risk prediction models often lack a justification for the sample size used. Only 17.8% (*n* = 27/152) of studies provided a justification, with the most common justification being the size of the available data.^1^ Dhiman et al.^7^ reported that only 8% (*n* = 5/62) of the studies using MLTs to develop prediction models had justified their sample sizes, with these studies having lower events per variable (EPV) than studies using regression-based models (median: 3.4, interquartile range (IQR): 1.1–19.1 versus median: 8, IQR: 7.1–23.5). The justifications of their sample sizes were either based on regression-based recommendations or the availability of data. Furthermore, Christodoulou et al.^8^ reported a median EPV of 8 (Range: 0.3–6697) across 71 studies on risk prediction using MLTs for binary outcomes. Wang et al*.*^9^ reported a median sample size of 475 (Range: 70–3184) with a median number of predictors of 22 (Range: 4–152) in their review of 18 studies using MLTs to predict various health outcomes following stroke. Additionally, Andaur Navarro et al.^1^ found that among 19 studies using MLTs for prediction model development with external validation, only three justified their sample sizes for external validation with all justifications based on data availability.

It is crucial to ensure that the sample size is adequate for both the development and validation of prediction models. Using a sample size that is too small is often one of the most common methodological flaws in studies using either logistic regression^10^ or a MLT.^7^ Recently, practical guidance has been developed for calculating the sample size required for the development^11^ and validation^12^ of logistic prediction models. Riley et al. proposed formulae to calculate the sample size required to, for example, limit model overfitting or to achieve a sufficiently small mean absolute prediction error (MAPE), among other model performance targets. The required sample size is context-specific and depends on factors such as the event proportion, the number of model parameters and the predictive strength of the model (*R*^2^/*C*-statistic).^11^ It has been suggested that these sample size calculations for logistic regression (fitted using maximum likelihood) may also be used to guide sample size calculations when using MLTs for risk prediction.^10^ In fact, some studies developing prediction models for binary outcomes using MLTs have applied Riley et al.'s formulae to determine the minimum sample size required for model development.^13–15^ However, Van der Ploeg et al.^16^ suggested that developing a prediction model with MLTs may require more than 10 times the EPV needed to achieve adequate discriminatory ability using logistic regression.

Our work was motivated by the emerging need for sample size guidance for developing and validating MLT-based clinical prediction models. It was important to investigate whether the current sample size recommendations for developing and validating clinical risk prediction models using logistic regression are applicable to or can be adapted for MLTs. Machine learning encompasses a wide range of techniques, and the choice of an appropriate method depends on the specific problem and the nature of the available data. In this study, we focused on ensemble tree-based methods which are commonly used to develop models for binary outcomes.^8^ Specifically, we considered bagged classification trees,^17^ RFs^18^ and gradient boosting machines.^19^ We conducted an extensive simulation study to evaluate the applicability of existing logistic regression-based sample size formulae for models using these MLTs. We considered a variety of data-generating scenarios typical in clinical data, including cases where there are interactions between predictors, and non-linear relationships between the outcome and predictors are present.

The datasets which were used as the basis for the simulations are introduced in Section 2. Section 3 provides a description of the ensemble tree-based MLTs. The design of the simulation study and its results are presented in Sections 4 and 5, respectively. Finally, the results from the simulation study are discussed and recommendations on the required sample size for using ensemble tree-based MLTs in the context of clinical prediction modelling are provided.

## 2 Datasets

We conducted simulations based on two datasets obtained from the National Institute for Cardiovascular Outcomes Research (NICOR). The purpose of using two different datasets was to examine how different features of the data may influence the sample size requirements for MLTs. One dataset contained non-linear associations between predictors and the outcome, and we introduced interactions between predictors in the other dataset. To handle missing values in predictor variables we applied multiple imputation by chained equations^20^ and then selected a single imputed dataset for both datasets for the simulations. The outcomes and predictor variables used from these data sources are listed in Tables S1 and S2 in the Supplemental Material.

### 2.1 Myocardial Ischaemia National Audit Project cohort

The first dataset was from the Myocardial Ischaemia National Audit Project (MINAP) and included 217,216 patients admitted to hospitals following an acute coronary syndrome (ACS). The binary outcome in-hospital death following hospital admission for an ACS had a prevalence of 6.28%. We considered 10 candidate predictors (4 continuous, 5 binary, 1 ordinal). Preliminary investigations using splines suggested that the relationships between the predictors and outcome were approximately linear.

### 2.2 Heart failure cohort

The second dataset included 54,081 patients admitted to hospital with heart failure (HF), where the binary outcome was 30-day mortality with a prevalence of 11.3%. It included 17 predictors (9 continuous, 8 binary). Investigations using splines suggested that a suitable regression model should account for non-linear relationships between the continuous predictors and the outcome.

## 3 Methods

In this section, we describe the MLTs used for both generating the data for simulations and model fitting.

### 3.1 Tree-based ensemble MLTs

In the context of binary classification, a classification tree (also known as a decision tree) is a supervised MLT based on an algorithm that builds a tree-like structure to make decisions. These algorithms aim to create a prediction rule based on a sequence of binary partitions of the data (i.e. tree). The partition at each step depends upon the answers to the previous yes/no questions in the sequence (e.g. Age > 66?). In a decision tree, a point where the data are split into two or more branches is called a *node*. The sequence terminates by predicting the class (event/no-event) label in the last binary partition for the subject (i.e. at the *terminal node*) when a stopping rule is triggered.^21^ Though single decision trees are computationally less expensive and easier to interpret, they may suffer from poor predictive accuracy.^22^ Tree-based ensemble methods, which combine the results of several classification trees can offer increased predictive accuracy over a single estimator. We considered bagging of classification trees, RFs, and gradient-boosting machines in this paper.^17–19^ Bagging (also known as bootstrap aggregating) fits decision trees repeatedly to bootstrapped datasets of the original training data.^17^ Each tree in the ensemble is trained independently and simultaneously on its corresponding bootstrap sample using all available predictors, then predictions from the individual trees are averaged. RFs improve upon bagging by reducing the correlation between trees in the ensemble by randomly choosing a subset of candidate predictors when building a tree, instead of using the full set of predictors.^18^ In both the bagging and RF algorithms, the final prediction is made through a majority voting scheme, where the predictions of individual trees are aggregated to determine the final predicted class. In other words, the final predicted class or outcome is the one with the largest number of votes across all fitted trees. The probability of a patient belonging to a particular class is estimated by the proportion of trees that predict that class for the patient.

Boosting algorithms construct sequential trees using information from previously grown trees, instead of training them in parallel. There is a range of boosting algorithms and they use different approaches to address the shortcomings of previous trees during the iterative process. We used stochastic gradient boosting machine (hereafter *boosting*), that takes a random subset with replacement from the original data to train each tree.^19,23^ For classification problems, boosting typically starts with one leaf node that predicts an initial value of the probability of the outcome based on the log-odds for every subject. The residuals calculated as the difference between the observed and predicted values are then used as the outcome for the following tree. By assigning more weight to misclassified observations with larger residuals, more accurate predictions can be made. The final prediction is made when the sum of the residuals is sufficiently small or when the maximum number of trees is reached.

We used the *caret* (classification and regression training) package in the R statistical programming language (R version 4.3.1, R Foundation for Statistical Computing, Vienna, Austria) to apply these methods.^24^ The *caret* package allows the user to train the model with a user-defined regression and classification method. We used the *treebag*, *rf* and *gbm* functions in the *caret* package (version 6.0-94) for bagging, RFs and boosting, respectively.

### 3.2 Hyperparameter tuning for MLTs

Hyperparameters are machine learning model parameters whose values are chosen before training the model and remain constant throughout the training process. The choice of appropriate hyperparameters can significantly affect the performance and behaviour of MLTs, and therefore hyperparameters are often tuned to optimise model performance.^25^ Different MLTs often use different hyperparameters according to their own unique characteristics such as model architecture, learning algorithm and assumptions. The choice of hyperparameters can depend on factors such as the type of model being used, model complexity, the distributions of the outcome and predictors, the size of the dataset, and the presence of noise or outliers.

The development and validation of predictive models using MLTs are typically conducted by splitting the original dataset into three subsets: training, validation, and testing datasets. The training dataset is used to develop the model, while the validation dataset is used for hyperparameter tuning. The testing dataset is used to evaluate the performance of a trained model in new data.

The R *caret* package provides an automated grid search for optimising hyperparameters during model training. We performed hyperparameter tuning using five-fold cross-validation on different combinations of training and validation sets by constructing a grid search of possible hyperparameter values, including the software's default values. For each combination of hyperparameter values within the grid, the training models were fitted to 10 simulated datasets across various simulation scenarios (described in Section 4). We determined the optimal hyperparameter values prior to running the full simulations to reduce the computational time to train the models. Brier scores were used as the primary performance metric with hyperparameter values producing the lowest Brier score considered to be optimal for each method within a simulation scenario.^26^ When there were no differences in the Brier score for possible hyperparameter values, we used the values that required the least model training time.

In the Supplemental Material, we provide a description of the hyperparameters, the range of hyperparameter values considered in the grid search, and the optimal values selected for the MINAP and HF datasets (Tables S3–S9).

### 3.3 Strategies for handling imbalanced dataset for MLTs

A dataset is said to be *imbalanced* when the number of individuals with and without the event are not represented equally.^27^ In prediction modelling, the issue of imbalanced data arises when predicting low/high prevalence events. The most prevalent and rarest outcome classes are referred to as the majority and minority classes, respectively. MLTs trained with imbalanced data have a tendency to underestimate the probabilities for the minority class.^28^ Therefore, various approaches have been developed to dealing with imbalanced data classification.^29^

A popular technique for dealing with imbalanced datasets is to utilise a form of resampling that creates artificial balanced samples for training the models.^30^ Depending on the strategy used to balance the class distribution, resampling techniques fall into three main groups: (i) *under-sampling by* randomly removing observations from the majority class until the class frequencies are equal,^31^ (ii) *over-sampling* by adding observations to the minority class by resampling from the class with replacement until the class frequencies are equal,^31^ and (iii) *hybrid methods* (techniques such as Synthetic Minority Over-Sampling Technique – SMOTE^32^ and Random Over-Sampling Examples – ROSE^33^) by under-sampling from the majority class and over-sampling by generating synthetic new observations in the minority class.

Cost-sensitive learning is another strategy to deal with the class imbalance problem which can yield superior predictive accuracy compared to using standard algorithms.^34^ This method relaxes the assumption that the misclassification costs of the events and non-events are the same. To ensure that the model accurately predicts the minority (i.e. events in our examples) class, cost-learning modifies the algorithm's training function by incorporating a hyperparameter which controls the ratio of class weights between the majority and minority classes.^35^ Specifically, increasing the weight of the minority class relative to the majority class helps the model to focus more on the minority class during training (e.g. 10:1, 20:1, 100:1, etc.).

Calibration correction is used to refine the predicted probabilities of a classification model, making them more aligned with the actual probabilities of class membership.^36^ In clinical prediction models, two main calibration models, namely Platt scaling^37^ and isotonic regression are used to obtain the calibrated probabilities.^38^ Platt scaling transforms probabilities using a logistic regression model with the estimated probabilities as the independent variable and the binary outcome as the dependent variable. Alternatively, isotonic regression adjusts the predicted probabilities by applying a monotonically increasing transformation to the original probabilities. If resampling techniques are used, calibrating probabilities may become even more important since resampling can introduce bias into the estimated probabilities.^39^

Due to the imbalanced nature of the MINAP and HF datasets, we assessed whether different techniques for handling imbalances improved the predictive performance and calibration of the MLT models. We applied a variety of approaches including resampling techniques, cost-sensitive models and probability calibration to 10 randomly generated simulated datasets across different simulation scenarios. No single strategy improved all of the performance measures simultaneously for any given scenario. The results are presented in the Supplemental Material (Tables S10–S27). Consequently, for the sample size evaluations we used the original models without applying any strategy to handle imbalance.

## 4 Simulation study

We used simulation to assess whether the existing sample size formulae for developing^11^ and validating^12^ clinical risk prediction models with logistic regression are appropriate for tree-based ensemble MLTs. The structure of this section is in line with ADEMP, a systematic framework for simulation studies proposed by Morris et al.^40^

### 4.1 Aims of the simulations

Aim 1. We assessed whether the sample size formula for targeting a small MAPE when developing a prediction model using logistic regression can be used to determine the sample size for developing tree-based ensemble MLT models.

Aim 2. We assessed whether the sample size formula for studies conducting external validation of prediction models based on logistic regression can be used to determine the sample size for the external validation of tree-based ensemble MLT models.

### 4.2 Data-generating mechanisms for simulating binary outcomes

We compared the performance of logistic regression and three MLTs for predicting binary outcomes using a simulation study. The data-generating mechanisms (DGMs) used to simulate the outcomes were based on each analysis model.^41^ In addition to DGMs that aligned with specific analysis models, we considered DGMs that introduced interactions between predictors. All DGMs were used for both aims.

*DGMs 1–4*: For both the MINAP and HF datasets, we considered each of the four analysis models under investigation as the basis for the DGM, namely (1) logistic regression with main effects (LR-ME for MINAP and LR-NL for HF, the NL indicating the inclusion of non-linear effects), (2) bagging, (3) RFs, and (4) boosting. For LR-NL, natural splines were used to model the continuous variables (LR-NL):

$$log\frac{P(Y=1|X)}{P(Y=0|X)}=\beta_{0}+\beta_{1}x_{1}+\ldots +\beta_{q}x_{q}+B_{1}NS(x_{q+1},\;df=4)+\ldots +B_{k-q}NS(x_{k},\;df=4)$$

where *q* is the number of categorical covariates and *B_1_, … B_k−q_* denote the 1 × 4 coefficient matrices for the continuous variables. We defined natural spline functions for the continuous predictors with the software default choice of four basis functions (i.e. 4 degrees of freedom), resulting in a model with 44 model parameters. The purpose of using this DGM was to assess how MLTs perform compared to logistic regression with respect to sample size, when the relationships between the outcome and some of the predictors are non-linear.

*DGM 5*: Additionally, for the MINAP dataset, we used a DGM based on a logistic regression model that included all main effects and two-way interactions, after categorising each continuous predictor into five equal-sized categories. There were 320 parameters in this DGM and we refer to it as a *neutral model* as its data structure does not match any of the analysis models. The purpose of using this DGM was to assess how MLTs perform compared to logistic regression with respect to sample size, when the true DGM has a complex structure that includes interaction terms.

### 4.3 Calculating sample sizes for development and external validation datasets

For Aim 1, the sample size for the development dataset, which will be referred to hereafter as *n_dev_*, was determined by the sample size formula proposed by Riley et al.,^11^ that targets MAPE (see Section 4.5 for full details), where MAPE is often used to evaluate the predictive accuracy of a prediction model and can be calculated for both logistic regression and MLTs. The sample size formula is given below:

(1) $$n_{dev}=exp\left(\frac{-0.508+0.259\ln\;(p)+0.504\ln\;(k)-\text{ln}(MAPE)}{0.544}\right),$$

where *p* is the anticipated outcome proportion (*p* ≤ 0.5) and *k* is the number of candidate predictor parameters. We set the value of *p* as that observed in the original data, and the value of *k* was based on the number of parameters considered for the logistic regression DGM. For the MINAP dataset, *p* and *k* were 0.063 and 10, respectively. For the HF dataset *p* was 0.113, and *k* was 44 accounting for the inclusion of non-linear terms and natural splines in the LR-NL DGM. We considered a range of target MAPE values for the MINAP (0.005, 0.007, 0.01, 0.015, 0.02) and HF (0.015, 0.02, 0.03) datasets to represent varying levels of predictive accuracy relative to the outcome prevalence.

For Aim 2, we used the formula proposed for calculating the sample size required for an external validation study to estimate a *C*-statistic with a desired level of precision, based on a model developed using logistic regression. This approach assumes that the linear predictor is normally distributed and requires information on the anticipated value of the *C*-statistic (C) and the outcome prevalence^12^:

(2) $${\hat{n}}_{valid}(C)=\frac{C-2T\left(\Phi^{-1}(C),\frac{1}{\sqrt{3}}\right)-C^{2}}{p(1-p)var_{req}(\hat{C})}\;,$$

where Φ is the cumulative distribution function of the standard normal distribution, *T* is Owen's *T*-function,^42^ and $var_{req}(\hat{C})$ is the target variance of the estimated *C*-statistic. For calculating $var_{req}(\hat{C})$ , we considered two values for the target standard error of $\hat{C}$ (0.010 and 0.025).

### 4.4 Design of the simulation study

The data-generation process was started by fitting a given model to the original dataset and predicting probabilities of the outcome for each subject (π_i_, i = 1, … , *N*). These were considered as the true probabilities and used to simulate binary outcomes as $y_{i}∼Bernoulli(\pi_{i}),\;i=1,\ldots N\;.$ These simulated outcomes were considered as the true outcomes of the full data for the simulation study.

For Aim 1, each simulation iteration involved drawing a development sample (with replacement) of size *n_dev_* from the observed covariates and simulated *y_i_* values. The sample size *n_dev_* was determined using Formula (1) for a range of target MAPEs. The outcomes were simulated using the five DGMs and five sample size scenarios for MINAP, and four DGMs and three sample size scenarios for HF. We then fitted each analysis model (i.e. LR-ME, LR-NL (only for HF dataset), bagging, RF and boosting) to the development data. The remaining subset of the full dataset that was not used for developing the model was used to validate the performance of the model, and for convenience, we refer to this as the validation sample for all methods considered, with *n_valid_* = *N* − *n_dev_*. The predicted outcome probabilities were calculated for the validation data ( ${\hat{\pi }}_{\text{i}}$ , i = 1, … , *n_valid_*) based on the fitted analysis models. The performance of the models was assessed using these fitted probabilities. For each scenario, this process was repeated 1000 times using development datasets from different DGMs.

For Aim 2, the true *C*-statistics and outcome proportion were calculated from the full data with the simulated outcomes. Then, the validation sample size, $n_{valid}$ , was calculated using Formula (2) for different values of the target SE(C). For each simulation iteration, a random sample of size $n_{valid}$ , with a given number of events ( $n_{events}$ ) was selected from the full data, and $SE(\hat{C})$ was calculated for the validation sample and compared to the pre-determined target SE(C). This process was repeated 1000 times using two different values for the target SE(C) for each DGM.

### 4.5 Performance measures and targets

#### 4.5.1 Aim 1: Development data

The primary performance measure of interest is MAPE. We assessed whether the MLT models can achieve the target MAPE under a range of development sample sizes, where MAPE quantifies a model's mean predictive accuracy and is estimated as

$$MAPE=\frac{1}{n_{valid}}\underset{i=1}{\sum }\;|{\hat{\pi }}_{i}\;-\pi_{i}|$$

Lower values of MAPE indicate greater predictive accuracy.

Model discrimination was assessed with the *C*-statistic (*C*). It is defined as the probability that a random individual with the event has a higher predicted probability than a random individual without the event and is estimated using

$$C=\frac{1}{n_{0,valid}n_{1,valid}}\underset{i:y_{i}=1}{\sum }\;\underset{j:y_{j}=0}{\sum }\;I({\hat{\pi }}_{i}>{\hat{\pi }}_{j})$$

where $n_{0,valid}$ and $n_{1,valid}$ are the numbers of observations in the validation set with $y_{i}$ *=* *0* and ${\text{y}}_{\text{j}}$ = 1, respectively, and *I(.)* is the indicator function.

The calibration of the predictions was examined to assess the agreement between the predicted and observed probabilities of the outcome. A variety of calibration measures were calculated; their stringency levels are defined by Van Calster et al*.*^43^ Calibration-in-the-large was calculated as the difference between the proportion of events in the validation data and the average of the predicted probabilities, and should ideally be zero. Values smaller than zero indicate systematic overestimation of the estimated probabilities, whereas values greater than zero indicate systematic underestimation. The calibration slope was calculated by regressing the observed outcome on the log-odds of the predicted probability of the outcome. A slope close to one indicates good calibration across a range of individuals or subgroups, whereas a slope greater (less) than one indicates underfitting (overfitting). The integrated calibration index (ICI) was calculated as the weighted average of the absolute differences between the observed outcome of a subject and the predicted probability for that subject, where the weights are the predicted probability values.^44^ The ICI ranges from 0 to 1, where lower values indicate better calibration. Another calibration measure, E90, was calculated as the 90th percentile of the absolute difference between the observed and predicted probabilities. It assesses the calibration of a model specifically in the region of the predicted probabilities where the model is most likely to make errors.^44^ The Brier score, which measures the deviation between the true outcome and predicted probability, was estimated as

$$Brier\;Score\;=\frac{1}{n_{valid}}\underset{i=1}{\sum }\;(y_{i}-{\hat{\pi }}_{i}\;{)}^{2}$$

The *C*-Statistic, Brier Score, E90 and calibration measures were computed using the *val.prob* function from the *rms* package in *R*.^45^

#### 4.5.2 Aim 2: Validation data

We estimated the standard error of $\hat{C},$ $SE(\hat{C})$ using DeLong's variance estimator.^46^

## 5 Results

### 5.1 R Shiny web application

The performance of the logistic regression and tree-based risk models for different data generation processes and sample sizes are presented in a freely accessible R Shiny web application (available from https://mlsamplesize.shinyapps.io/Sample_size_ML/). The web application provides a comparison of methods across a range of performance measures, assessing predictive accuracy and calibration through easy-to-read boxplots.

**Table 1.** Median (25th–75th percentile) of the MAPE (×100) for the MINAP dataset over 1000 simulations.

						Analysis model	
DGM	*n_dev_*	EPV	Target MAPE (×100)	LR-ME	Bagging	RF	Boosting
LR-ME	1182	7.5	2.0	**1.56** (**1.36–1.79)**	4.99 (4.76–5.21)	3.61 (3.41–3.78)	2.81 (2.54–2.98)
	2005	12.6	1.5	**1.22** (**1.00–1.41)**	4.78 (4.54–4.95)	3.43 (3.31–3.52)	2.40 (2.24–2.53)
	4225	26.6	1.0	**0.80** (**0.68–0.96)**	4.43 (4.30–4.57)	3.26 (3.17–3.34)	2.06 (1.93–2.18)
	8138	51.3	0.7	**0.59** (**0.53–0.69)**	4.25 (4.16–4.34)	3.22 (3.17–3.30)	1.87 (1.78–1.94)
	15,105	95.1	0.5	**0.41** (**0.36–0.47)**	4.16 (4.11–4.24)	3.29 (3.24–3.34)	1.76 (1.7–1.83)
Bagging	1182	7.5	2.0	6.88 (6.62–7.04)	7.69 (7.41–7.94)	**6.59** (**6.46–6.75)**	6.96 (6.74–7.17)
	2005	12.6	1.5	6.85 (6.65–6.97)	7.60 (7.40–7.79)	**6.39** (**6.28–6.51)**	6.87 (6.76–7.06)
	4225	26.6	1.0	6.78 (6.67–6.92)	7.40 (7.28–7.54)	**6.08** (**6.02–6.14)**	6.83 (6.73–6.97)
	8138	51.3	0.7	6.75 (6.69–6.82)	7.22 (7.15–7.34)	**5.84** (**5.80–5.86)**	6.79 (6.74–6.87)
	15,105	95.1	0.5	6.75 (6.68–6.82)	7.04 (6.97–7.14)	**5.62** (**5.59–5.64)**	6.78 (6.71–6.84)
RF	1182	7.5	2.0	6.47 (6.17–6.68)	7.41 (7.14–7.71)	**6.23** (**6.08–6.41)**	6.55 (6.24–6.75)
	2005	12.6	1.5	6.35 (6.22–6.51)	7.25 (7.05–7.44)	**5.96** (**5.90–6.13)**	6.42 (6.26–6.60)
	4225	26.6	1.0	6.33 (6.26–6.52)	7.05 (6.91–7.16)	**5.70** (**5.65–5.77)**	6.34 (6.26–6.50)
	8138	51.3	0.7	6.34 (6.26–6.43)	6.92 (6.84–7.01)	**5.45** (**5.42–5.51)**	6.34 (6.26–6.43)
	15,105	95.1	0.5	6.32 (6.27–6.38)	6.74 (6.68–6.81)	**5.24** (**5.22–5.28)**	6.35 (6.26–6.41)
Boosting	1182	7.5	2.0	2.38 (2.20–2.62)	5.19 (4.92–5.40)	3.58 (3.39–3.72)	**2.37** (**2.16–2.61)**
	2005	12.6	1.5	2.09 (1.97–2.25)	4.85 (4.67–5.07)	3.38 (3.27–3.48)	**1.89** (**1.70–2.10)**
	4225	26.6	1.0	1.93 (1.87–2.00)	4.59 (4.53–4.69)	3.24 (3.16–3.32)	**1.49** (**1.34–1.62)**
	8138	51.3	0.7	1.84 (1.79–1.89)	4.44 (4.37–4.53)	3.20 (3.15–3.25)	**1.25** (**1.17–1.32)**
	15,105	95.1	0.5	1.79 (1.76–1.82)	4.38 (4.32–4.44)	3.29 (3.22–3.34)	**1.01** (**0.94–1.07)**
Neutral	1182	7.5	2.0	**2.83** (**2.65–3.04)**	4.77 (4.55–5.01)	3.47 (3.36–3.61)	3.06 (2.89–3.21)
	2005	12.6	1.5	**2.63** (**2.54–2.73)**	4.57 (4.35–4.80)	3.30 (3.21–3.44)	2.79 (2.64–2.93)
	4225	26.6	1.0	**2.46** (**2.41–2.51)**	4.33 (4.22–4.43)	3.14 (3.04–3.23)	**2.46** (**2.39–2.56)**
	8138	51.3	0.7	2.39 (2.36–2.41)	4.17 (4.08–4.28)	3.10 (3.04–3.19)	**2.28** (**2.22–2.36)**
	15,105	95.1	0.5	2.35 (2.33–2.37)	4.11 (4.05–4.17)	3.16 (3.08–3.23)	**2.15** (**2.11–2.21)**

Bold values indicate the analysis models with the smallest median MAPE within a particular DGM and sample size scenario. EPV: event per variable, DGM: data-generating mechanism; MAPE: mean absolute prediction error; MINAP: Myocardial Ischaemia National Audit Project; LR-ME: logistic regression with main effects; RF: random forest.

### 5.2 Sample size assessments for model development

#### 5.2.1 MINAP dataset

**Figure 1.**
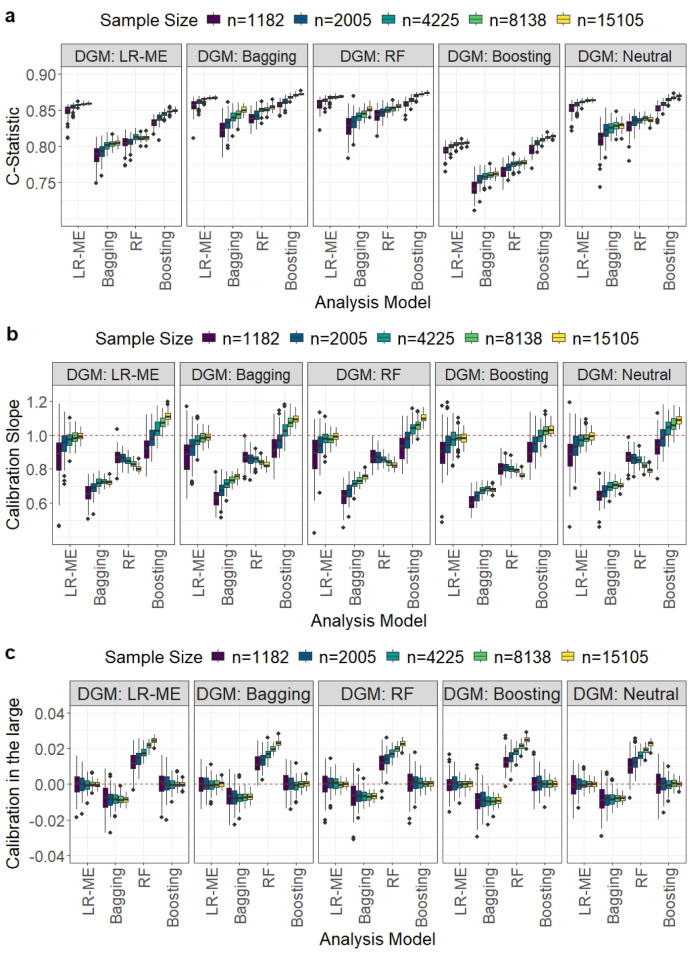
Comparison of model performance in terms of the *C*-statistic, calibration slope, and calibration-in-the-large for the Myocardial Ischaemia National Audit Project (MINAP) dataset. Each boxplot displays the distribution of a given performance metric across the 1000 simulation iterations for different development sample size scenarios when a particular data-generating mechanism (displayed in header) and analysis model were used.

The results for the various performance measures for the four analysis models under five different sample sizes and DGMs are reported in Table 1 and Figure 1.

##### 5.2.1.1 Matching DGMs and analysis models

As expected, the value of MAPE decreased with increasing sample size for all models. With the exception of bagging, the best performance for each DGM was achieved by the matching analysis model. None of the ML models achieved the target MAPE at the recommended sample sizes. Boosting needed at least twice the sample size of LR-ME to achieve the target MAPE of 2% and 1.5%. To achieve a smaller target MAPE of 1%, boosting required approximately 3.5 times more data than the recommended sample size. Bagging and RF were not close to achieving the target MAPE, even if the recommended sample size was increased by 15 times with bagging performing the worst (Table 1).

It may be noted that bagging and RF calculate the predicted probabilities of the outcome as the proportion of trees that voted for the event class to the total number of trees in the ensemble; when none of the trees vote for the event class the predicted probability of the event is zero. In our simulations, bagging and RF predicted most of the probabilities to be zero. This may be due to the low prevalence of events in the MINAP dataset; furthermore, the small number of predictors may have decreased the diversity of the trees. To calculate the *C*-statistic, we replaced the 0 and 1 predictions with 0.0001 and 0.9999, respectively. We also note that for the boosting DGM, the estimated *C*-statistic was lower than that for the other DGM scenarios for all analysis models. The calculated *C*-statistic increased with sample size for all analysis models. The LR-ME and boosting analysis models (true *C*-statistic of 0.86 and 0.82, respectively) outperformed bagging and RF (true *C*-statistic values of 0.96) for all recommended sample sizes (Figure 1(a)). However, boosting required substantially larger sample sizes to reach close to the corresponding true *C*-statistic values.

For the recommended sample sizes, LR-ME and boosting resulted in median calibration slopes close to unity and calibration-in-the-large values very close to zero (Figures 1(b) and (c)). In contrast, both calibration slope and calibration in-the-large were worse for RF and worst for bagging. Observations with estimated probabilities of 0 and 1 were excluded from the calculation of the RF and bagging calibration measures and the exclusion of many data points may have contributed to their poor performances.

The results for the Brier score, ICI and E90 can be found in the R Shiny app. For the recommended sample sizes, the LR-ME and boosting models had E90 and ICI values closer to zero than that of the other methods. Additionally, the LR-ME and boosting models produced smaller Brier scores than the other MLT models for the recommended sample sizes.

##### 5.2.1.2 Non-matching DGMs and analysis models

For the LR-ME DGM, boosting was able to achieve a target MAPE of 2% with at least four times the recommended sample size. For the boosting DGM, LR-ME approached the target MAPE of 2% when the sample size was more than twice the recommended size. Neither LR-ME nor boosting attained a MAPE value much lower than 2% even when the recommended sample size was increased more than 12 fold. However, both models outperformed RF and bagging for all non-matching DGMs except for the bagging DGM where RF performed best. Bagging and RF failed to achieve MAPE values close to the target even with substantial increases in the recommended sample size. For DGM-Neutral, boosting and LR-ME were only able to achieve a target MAPE close to 2% when the recommended sample size was increased by more than 12 times.

For the *C*-statistic the LR-ME and boosting performed better than bagging and RF for the recommended sample sizes with boosting showing greater improvement with increasing sample size.

LR-ME resulted in median calibration slopes ≥ 0.9 with an IQR including one and calibration-in-the-large very close to zero for the recommended sample sizes (Figure 1(b) and (c)), with performance improving with increasing sample size. Boosting tended to result in slight underfitting of the model for large sample sizes (>8000), with median calibration slopes ranging from 1.06 to 1.11. However, the calibration-in-the-large values were close to zero. Bagging and RF performed poorly for the calibration measures and increasing the sample size did not improve the results for RF.

While LR-ME produced the lowest Brier scores for smaller sample sizes, boosting produced the lowest Brier scores for the larger sample sizes. Both LR-ME and boosting resulted in E90 and ICI values close to zero.

#### 5.2.2 HF dataset

**Figure 2.**
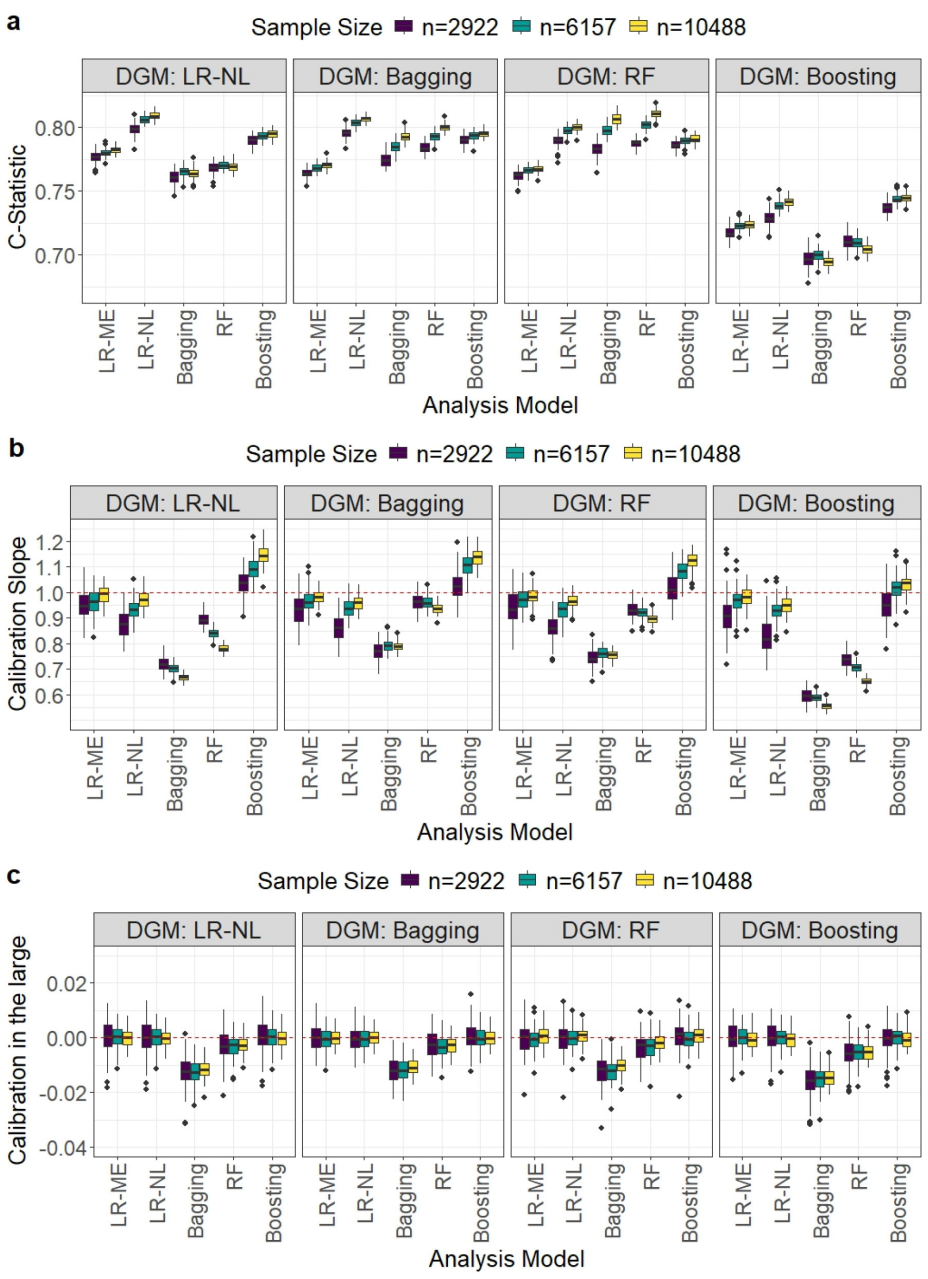
Comparison of model performance in terms of the *C*-statistic, calibration slope, and calibration-in-the-large for the HF dataset. Each boxplot displays the distribution of a given performance metric across the 1000 simulation iterations for different development sample size scenarios when a particular DGM and analysis model were used. HF: heart failure; DGM: data-generating mechanism.

In contrast to the MINAP dataset, the HF dataset had non-linear relationships between the outcome and predictors as well as a higher number of predictors and outcome prevalence. The performance of the five analysis models under three different sample sizes and four DGMs is reported in Table 2 and Figure 2.

**Table 2.** Median (25th–75th percentile) of the MAPE (×100) for the heart failure dataset over 1000 simulations.

						Analysis model		
DGM	*N*	EPV	Target MAPE (×100)	LR-ME	LR-NL	Bagging	RF	Boosting
LR-NL	2922	7.5	3	4.29 (4.14–4.44)	**2.79** (**2.54–3.01)**	5.94 (5.78–6.19)	4.97 (4.84–5.11)	3.57 (3.38–3.75)
	6157	15.8	2	4.09 (3.99–4.2)	**1.88** (**1.75–2.07)**	5.82 (5.68–5.9)	4.84 (4.75–4.93)	3.35 (3.24–3.45)
	10,448	26.8	1.5	4.04 (3.99–4.11)	**1.45** (**1.36–1.54)**	5.93 (5.82–6.02)	5.04 (4.98–5.10)	3.32 (3.20–3.42)
Bagging	2922	7.5	3	11.77 (11.49–11.92)	**10.55** (**10.33–10.82)**	11.51 (11.21–11.68)	11.09 (10.89–11.31)	10.90 (10.65–11.12)
	6157	15.8	2	11.72 (11.56–11.88)	**10.48** (**10.29–10.61)**	11.01 (10.79–11.16)	10.71 (10.53–10.82)	10.99 (10.80–11.13)
	10,448	26.8	1.5	11.70 (11.6–11.79)	10.39 (10.29–10.52)	10.49 (10.41–10.63)	**10.21** (**10.14–10.33)**	11.02 (10.87–11.13)
RF	2922	7.5	3	10.60 (10.44–10.77)	**9.51** (**9.33–9.75)**	10.60 (10.41–10.81)	10.15 (9.9–10.31)	9.74 (9.53–9.96)
	6157	15.8	2	10.55 (10.45–10.71)	**9.40** (**9.29–9.55)**	10.21 (10.09–10.37)	9.74 (9.63–9.85)	9.79 (9.69–9.94)
	10,448	26.8	1.5	10.50 (10.40–10.62)	**9.35** (**9.26–9.44)**	9.79 (9.73–9.87)	**9.35** (**9.26–9.43)**	9.81 (9.72–9.94)
Boosting	2922	7.5	3	3.87 (3.73–3.99)	3.26 (3.06–3.48)	6.28 (6.12–6.49)	4.87 (4.73–4.99)	**2.49** (**2.33–2.71)**
	6157	15.8	2	3.59 (3.48–3.67)	2.43 (2.33–2.53)	6.12 (6.03–6.26)	4.85 (4.77–4.94)	**2.00** (**1.93–2.08)**
	10,448	26.8	1.5	3.52 (3.44–3.60)	2.09 (2.02–2.16)	6.32 (6.23–6.43)	5.10 (5.02–5.16)	**1.79** (**1.69–1.90)**

Bold values indicate the analysis models with the smallest median MAPE within a particular DGM and sample size scenario. EPV: event per variable; DGM: data-generating mechanism; MAPE: mean absolute prediction error; LR-NL: logistic regression including non-linear effects; RF: random forest.

LR-ME did not have a matching DGM. For the other matching DGM and analysis model scenarios, LR-NL and boosting achieved the target MAPEs at the recommended sample sizes (based on LR-NL) (Table 2). In the non-matching scenarios, none of the methods achieved the target MAPE values with LR-ME performing similarly to the MLT models and LR-NL producing the smallest MAPE values for all sample sizes.

For matching DGMs, LR-NL achieved the highest *C*-statistic values, attaining values close to the true value of 0.81 (Figures 2(a)). For non-matching DGMs, LR-NL performed the best followed by boosting at the recommended sample sizes.

For matching DGMs, LR-NL, RF and boosting produced median calibration slopes close to 0.9 with IQRs that include zero and calibration in the large values close to zero (Figures 2(b) and (c)) at the recommended sample sizes. These calibration measures did not improve with increasing sample size for RF and boosting showed some under-fitting for large sample sizes. For non-matching DGMs, LR-ME, LR-NL and boosting performed better than bagging and RF, with bagging showing the worst performance at the recommended sample sizes. It may be noted that RF demonstrated comparatively better performance for the calibration measures compared to that observed for the MINAP dataset.

For matching DGMs, LR-NL produced the smallest Brier score at the recommended sample size. For non-matching DGMs, LR-NL and boosting achieved smaller Brier scores than bagging and RF. The only exception was for the bagging DGM, as RF tended to produce the smallest Brier scores at the recommended sample size.

All analysis models except bagging produced similar E90 and ICI values for all sample sizes and DGMs.

### 5.3 Sample size assessments for the validation dataset

Two different precision levels for the *C*-statistic were considered to calculate sample sizes for the external validation data for each DGM. The validation samples were randomly selected from the full data for this investigation. It may be noted that these sample size calculations assume normality of the linear predictor. However, it is highly likely that the distribution of the linear predictor from the MLT models will not be normal.

#### 5.3.1 MINAP dataset

The distributions of the linear predictor for the various DGMs are presented in Figure S1 in the Supplemental Material. The boosting DGM showed some skewness, and the bagging and RF DGMs showed considerable departures from normality, while the Neutral DGM conformed to a normal distribution. The validation sample size requirements and the mean *C*-statistics obtained for the validation samples are presented in Table 3, along with their standard errors for the two target SE(C) scenarios. The sample size for the external validation data depends on the value of the *C*-statistic with higher values of the *C*-statistic resulting in smaller sample size requirements. The smallest *C*-statistic value was obtained for the boosting DGM which is consistent with the results seen for the development data. The true values of the *C*-statistics were larger for bagging and RF (0.960 for bagging, 0.963 for RF). For all DGMs, the mean estimated standard error of the *C*-statistic achieved the target SE(C) value with the sample sizes calculated using the proposed formula despite the violation of the normality assumptions.

**Table 3.** Summary of the validation sample size results for the MINAP dataset.

			Calculated with Pavlou et al.'s formula^12^		Calculated across 1000 simulations	
DGM	True C	Target SE(C)	*n*_valid_	*n*_events_	$\hat{C}$	$SE(\hat{C})$
LR-ME	0.862	0.010	5525	348	0.864	0.007
		0.025	880	55	0.865	0.018
Bagging	0.960	0.010	829	55	0.952	0.009
		0.025	133	9	0.950	0.020
RF	0.963	0.010	1077	70	0.963	0.007
		0.025	173	12	0.962	0.018
Boosting	0.817	0.010	7325	462	0.818	0.008
		0.025	1172	74	0.811	0.019
Neutral	0.883	0.010	4632	287	0.883	0.007
		0.025	742	46	0.877	0.018

MINAP: Myocardial Ischaemia National Audit Project; DGM: data-generating mechanism; LR-ME: logistic regression with main effects; RF: random forest.

#### 5.3.2 HF dataset

The distribution of the linear predictor for the boosting DGM was slightly skewed, whereas the bagging and RF DGMs had skewed and bimodal distributions (Supplemental Material Figure S2) for the HF dataset.

Similar to the findings for the MINAP dataset, the target SE(C) was achieved for all the DGMs with the validation sample size calculated using the proposed formula (Table 4).

**Table 4.** Summary of the validation sample size results for the heart failure dataset.

			Calculated with Pavlou et al.'s formula^12^		Calculated across 1000 simulations		
DGM	True C	Target SE(C)	*n*_valid_	*n*_events_	$\hat{C}$	$SE(\hat{C})$
LR-NL	0.810	0.010	4432	502	0.810	0.008
		0.025	719	80	0.810	0.020
Bagging	0.905	0.010	1389	166	0.904	0.010
		0.025	228	27	0.905	0.022
RF	0.896	0.010	1980	227	0.896	0.008
		0.025	300	34	0.878	0.021
Boosting	0.753	0.010	5656	919	0.752	0.009
		0.025	919	104	0.752	0.022

DGM: data-generating mechanism; LR-NL: logistic regression including non-linear effects; RF: random forest.

## 6 Discussion

Machine learning models are increasingly being used by researchers for clinical prediction modelling. However, many of these researchers do not justify their chosen sample sizes.^1^ In this study, we investigated whether the existing MAPE-based approach for calculating the sample size for the development of logistic prediction models^10,11^ could be used or adapted for ensemble tree-based MLTs. This approach, which uses information on the number of predictors and outcome prevalence, is popular and available in widely-used software. We considered a range of sample sizes corresponding to different target MAPE values and investigated whether bagging, RFs, boosting and logistic regression models could achieve these targets. Additionally, we examined model performance using various metrics that capture different aspects of predictive ability, such as calibration and discrimination. We also investigated whether a precision-based sample size calculation for the external validation of a predictive model with a binary outcome, based on the standard error of the *C*-statistic,^12^ applies to models developed with these MLTs.

### 6.1 Simulation findings and recommendations

We performed simulations based on two large cardiovascular datasets with different outcome prevalences, numbers of predictors and levels of data complexity. In one dataset, interactions between the predictors were introduced, while the other had non-linear associations between the outcome and predictors. Thus both exhibited complex data structures where MLT models might be expected to perform better than conventional logistic regression models.

For the dataset with fewer (10) predictors and a lower prevalence with only linear associations between the outcome and the predictors, bagging and RF did not achieve the target MAPE or satisfactory values of the calibration measures with the recommended sample sizes. That was the case even when the DGM matched the analysis model and the recommended sample size was increased by more than 12-fold. Boosting achieved the target MAPE for a matching DGM with a sample size at least 2–3 times larger than that recommended and attained satisfactory values for the other performance measures. For the neutral DGM representing a complex data structure with all main effects and two-way interactions of the predictors, logistic regression including only main effects and boosting achieved MAPEs values close to the target with a 12-fold increase in the recommended sample size, while bagging and RF failed to achieve the target. For the dataset which had a larger (17) number of predictors, higher prevalence and non-linear associations between the outcome and predictors, the logistic regression model that accounted for non-linearities through natural splines performed the best, followed by boosting, at the recommended sample sizes with matching DGMs and analysis models. Logistic regression excluding the non-linear terms performed better overall than bagging and RF for the recommended sample sizes based on LR-NL. Bagging performed the worst in all scenarios for both datasets even with large sample sizes and matching DGMs.

Consistent with our findings, recent simulation studies have shown superior performance of boosting over other MLTs for developing clinical prediction models with binary^41^ and survival^47^ outcomes. Austin et al.^41^ used six different DGMs and found that logistic regression and boosting generally outperformed other MLTs across a range of performance measures. Their simulations were based on two datasets with EPVs of 56 and 96 but they did not vary the sample size. Infante et al*.*^47^ compared the predictive performance of MLTs relative to regression-based methods for developing a survival prediction model across different sample size scenarios. They reported that boosting algorithms achieved comparable performance to regression-based models using the same sample size. However, RFs needed at least 2 to 3 times the sample size of the conventional regression-based survival models to achieve the same values of the Brier score.

As summarised in Table 5, our study suggests that MLT models may require significantly larger sample sizes than those recommended by the logistic regression-based formula for achieving a target MAPE in model development. However, it might be possible to apply existing sample size formula for logistic regression to boosting if the recommended sample sizes are inflated to some extent. One approach for inflation could be to increase the number of parameters that goes into the calculation of the sample size. This could be achieved, for example, by categorising the continuous predictors into a number of percentile-based categories and adding terms for two-way interactions between all the predictors. Alternatively, one could account for a number of spline terms for each continuous predictor in the sample size calculation. This approach effectively accounts for the fact that boosting aims to capture complex patterns in the data and the number of parameters in an equivalent logistic regression model would be much higher than a regression model with only main effects. These types of inflation should also allow the flexibility of fitting a logistic regression model with nonlinear terms and interactions to a complex data structure and achieve adequate levels of predictive performance. However, one may also opt to use a simulation-based sample size calculation using a suitable DGM analogous to the approach of Pavlou et al.^48^ for logistic regression.

**Table 5.** Summary of the sample size requirements for model development using MLTs in comparison to the recommended sample size based on logistic regression to achieve a target MAPE.

Scenario	True DGM	Best performing method(s)	Sample size considerations compared to recommended sample size for LR to achieve target MAPE	Notes
1	Matching	LR, Boosting, RF	Boosting required 2–3 times	RF failed to achieve target MAPE even with a 12-fold increase in sample size.
	Non-matching			
	LR	Boosting	At least 4 times	None of the models achieve a MAPE value much lower than 2%.
	Bagging	RF	Did not achieve target MAPE	
	RF	LR	Did not achieve target MAPE	
	Boosting	LR	At least 2 times	
	Neutral	LR, Boosting	More than 12 times.	
2	Matching	LR-NL, Boosting	Boosting required the same sample size as LR-NL	
	Non-matching			
	LR-NL	Boosting	More than 4 times	
	Bagging	LR-NL, RF	None of the models achieve a MAPE value lower than 10%.	RF performed better only for the largest sample size scenario (EPV = 26.8)
	RF	LR-NL	None of the models achieve a MAPE value lower than 9%.	
	Boosting	LR-NL	At least 2 times	

Scenario 1: MINAP dataset (number of predictors: 10, *k* = 10, *p* = 0.63, only linear associations). Scenario 2: HF dataset (number of predictors: 17, *k* = 44, *p* = 0.113, non-linear associations). EPV: events per variable; MINAP: Myocardial Ischaemia National Audit Project; HF: heart failure; LR: logistic regression with only main effects; LR-NL: logistic regression including non-linear effects; RF: random forest.

The sample size evaluation for external validation of prediction models revealed that the target standard error of the *C*-statistic was achieved for all DGMs when the sample size was estimated using the precision-based formula^12^ even when the assumption of normality for the distribution of the linear predictor was violated for the MLT DGMs. Thus, this formula may be used to calculate the sample size for external validation studies using MLT models with binary outcomes.

### 6.2 Limitations and implications for future research

This study has some limitations. First, our simulations were based on two real-life cardiovascular datasets with a relatively small number of predictors, all of which were strongly associated with the outcome. Previous simulations that have evaluated the sample size formula for model development that was investigated in this paper have shown that its accuracy decreases for models with high prognostic strength—for instance, when the *C*-statistic exceeds 0.8, as observed in our datasets.^48^ A potential explanation for the relatively poor performance of bagging and RF in our study may be linked to the strength of the predictors, as optimal performance in ensemble tree-based machine learning classifiers requires low inter-tree correlations.^49^ This is because one of the key principles behind these ensemble models is to reduce overfitting and improve generalisation by combining the predictions from multiple trees. However, when there are strong predictors in the dataset, bagging tends to generate similar trees.^17^ This means that the strongest predictors will be chosen as the root node for the first split, and all of the trees will subsequently follow with similar splits. When these highly correlated trees are aggregated, the reduction in the variance of the predictions is smaller compared to a scenario with uncorrelated trees. RF addresses this problem by randomly selecting candidate predictors for each tree. However, a large number of predictors is necessary to improve prediction accuracy and reduce generalisation error for RF models.^18^ Second, the prevalence of the outcome in both datasets was low. In machine learning, having an imbalance in the data where one outcome class is a small minority may distort the predicted probabilities and predictive accuracy.^50,51^ However, health outcomes with low prevalences are common in many real-world problems. We attempted to address the class imbalance problem using various techniques, but none of them showed a consistent improvement across all performance measures. Further research is needed to evaluate the sample size needs of machine learning models in relation to outcome prevalence and techniques for solving the class imbalance problem. Another limitation was that hyperparameter selection was performed prior to the simulations by training the models on just the first 10 simulated datasets across various simulation scenarios. While this approach may not fully capture dataset-specific optimisations, we aimed to reduce the training time as hyperparameter tuning for MLTs can be complex and computationally demanding.

The RF model showed overfitting (CS < 1) and increasing the sample size did not improve calibration performance. We recommend caution when using calibration measures such as the calibration slope and calibration in-the-large with tree-based MLTs because these models can make predictions of exactly 0 or 1, which is typically not the case with logistic regression. Moreover, the skewed distribution of the linear predictor for bagging and RF may have additionally contributed to the poor performance of these calibration measures. More methodological work is needed to investigate the appropriateness of these calibration measures when using tree-based MLTs. When calculating the sample size for model development with RF, one may need to choose alternative performance measures that would be more suitable for assessing the predictive ability of these types of models.

A practical limitation of this study is that while our findings provide broad guidance on sample size considerations for model development and validation studies using MLTs and suggest ways to adapt the existing sample size formulas for logistic regression to account for complex data features, they do not offer readily applicable, method-specific sample size formulas for practitioners. Thus, it highlights the need for further investigations to establish robust sample size formulae that ensure reliable predictions across different MLTs and datasets. Additionally, the use of more real-world datasets with different characteristics may help in confirming our findings regarding the suitability of the logistic regression-based formulas for determining the sample sizes for model development and validation with MLTs.

## Supplemental Material

sj-docx-1-smm-10.1177_09622802251338983 - Supplemental material for Evaluating the sample size requirements of tree-based ensemble machine learning techniques for clinical risk prediction Supplemental material, sj-docx-1-smm-10.1177_09622802251338983 for Evaluating the sample size requirements of tree-based ensemble machine learning techniques for clinical risk prediction by Oya Kalaycıoğlu, Menelaos Pavlou, Serhat E Akhanlı, Mark A de Belder, Gareth Ambler and Rumana Z Omar in Medical Research
