# Supplementary material for: Evaluating the sample size requirements of tree-based ensemble machine learning techniques for clinical risk prediction
Source: Stat Methods Med Res. 2025 May 14;34(7):1356–72. doi: 10.1177/09622802251338983 (PMC12308042; doi:10.1177/09622802251338983)
Supplement: sj-docx-1-smm-10.1177_09622802251338983 - Supplemental material for Evaluating the sample size requirements of tree-based ensemble machine learning techniques for clinical risk prediction [file sj-docx-1-smm-10.1177_09622802251338983.docx]

**Supplementary Material**

**1. The list of the outcome and predictor variables**

**Table S1.** Summary of variables used in MINAP dataset (N=217,216)

| **Predictors** |  |  |
| --- | --- | --- |
| ***Continuous Variables*** | **Mean (SD)** | **Range** |
| Age (years) | 68.5 (13.9) | 19-110.5 |
| Heart Rate (bpm) | 80.1 (20.9) | 30-320 |
| Systolic Blood Pressure (mmHg) | 137.6 (27.9) | 31.23-250 |
| Creatinine (μmol/L) | 1.1 (0.7) | 0.21-11.3 |
| ***Ordinal variable*** | **Median (Q_1_-Q_3_)** | **Range** |
| Killip Score | 1 (1-1) | 1-4 |
| ***Binary variables*** | **N** | **%** |
| Cardiac Arrest, *yes* | 8,750 | 4.03 |
| Elevated biomarkers, *yes* | 202,327 | 93.1 |
| ST Deviation, *yes* | 115,321 | 53.1 |
| Non-white | 18,371 | 8.5 |
| Nstemi, *yes* | 120,698 | 55.6 |
| **Outcome** | **N** | **%** |
| In hospital mortality | 13,649 | 6.3 |

**Table S2.** Summary of variables used in Heart Failure dataset (N=54,081)

| **Predictors** |  |  |
| --- | --- | --- |
| ***Continuous Variables*** | **Mean (SD)** | **Range** |
| Age (years) | 78.3 (12.3) | 18-109.1 |
| Serum Creatinine at discharge (μmol/L) | 128.1 (80.6) | 30-1405 |
| Serum Sodium at discharge (mEq/L) | 137.5(5.0) | 100-160 |
| Serum Potassium at discharge (mmol/L) | 4.3 (0.6) | 1.7-7.9 |
| Urea at discharge (mg/dL) | 12.8 (10.0) | 2-100 |
| Systolic blood pressure at admission (mmHg) | 134.3 (27.1) | 60-284 |
| Heart Rate at admission (bpm) | 86.9 (23.0) | 30-270 |
| Hemoglobin at discharge (g/dL) | 12.0 (2.1) | 5-20 |
| eGRF (estimated glomerular filtration rate) | 53.8 (25.6) | 2.82-284 |
| ***Binary variables*** | **N** | **%** |
| Gender, *Female* | 24,728 | 45.07 |
| Diabetes, *yes* | 18,376 | 34.00 |
| Chronic obstructive pulmonary disease, *yes* | 10,037 | 18.60 |
| [Ischemic Heart Disease, *yes*](https://www.ncbi.nlm.nih.gov/books/NBK209964/#:~:text=Ischemic%20heart%20disease%2C%20also%20called,blood%20to%20the%20heart%20muscle.) | 21,732 | 40.20 |
| Valve disease, yes | 14,198 | 26.30 |
| New York Heart Association (NYHA), *Class I/II* | 11,429 | 78.90 |
| Peripheral Oedema, *Moderate/Severe* | 29,205 | 54.00 |
| Atrial Fibrillation, *yes* | 24,448 | 45.20 |
| **Outcome** | **N** | **%** |
| 30-days mortality | 6,101 | 11.3 |

**2. Description and optimal hyperparameter values for each simulation scenario.**

**Table S3.** Machine learning models and their corresponding hyperparameters.

| **ML Model** | **Hyperparameter** | **Description of Hyperparameter** | **Hyperparameter values considered in grid search** |
| --- | --- | --- | --- |
| Bagging | Nbagg | Number of trees in the ensemble | 25, 50, 100 |
|  | Maxdepth | Maximum number of nodes between the root and the final nodes | 1, 2, 3, 4, 5 |
|  | Nodesize | Minimum number of observations in a terminal node | 1, 2, 3, 4, 5 |
| Random Forest | Ntrees | Number of trees in the forest | 100, 500, 1000 |
|  | Mtry | Number of randomly sampled predictors to be used in each tree | 1, 2, 3, 4, 5 |
|  | Nodesize | Minimum number of observations in a terminal node | 1, 2, 3, 4, 5 |
| Boosting | n.trees | Number of trees in the sequence | 100, 500, 1000 |
|  | interaction.depth | Maximum nodes per tree | 1, 3, 5 |
|  | shrinkage | Rate that controls how fast the model is learning from previous models | 0.01, 0.05, 0.10 |
|  | n.minobsinnode | Minimum number of observations allowed in the trees’ terminal nodes | 3, 5, 10 |

**Hyperparameter values and their associated Brier’s score for each scenario**

## **For MINAP Dataset**

**Table S4.** Hyperparameter values used for each scenario when analysis model is Bagging.

|  |  | **Hyperparameter values** | | |  |
| --- | --- | --- | --- | --- | --- |
| **DGM** | **Sample Size** | **Nbagg** | **Maxdepth** | **Nodesize** | **Median Brier Score (IQR)** |
| LR-ME | 1182 | 100 | 1 | 2 | 0.0515 (0.0491-0.0535) |
|  | 2005 | 100 | 3 | 1 | 0.0540 (0.0519-0.0551) |
|  | 4225 | 100 | 3 | 3 | 0.0530 (0.0525-0.0537) |
|  | 8138 | 100 | 2 | 2 | 0.0525 (0.0523-0.0528) |
|  | 15105 | 100 | 1 | 3 | 0.0522 (0.0521-0.0523) |
| LR-Neutral | 1182 | 100 | 2 | 3 | 0.0542 (0.0518-0.0567) |
|  | 2005 | 100 | 1 | 3 | 0.0535 (0.0524-0.0549) |
|  | 4225 | 100 | 2 | 3 | 0.0528 (0.0520-0.0535) |
|  | 8138 | 100 | 2 | 2 | 0.0521 (0.0517-0.0525) |
|  | 15105 | 100 | 1 | 2 | 0.0520 (0.0518-0.0522) |
| Bagging | 1182 | 100 | 1 | 2 | 0.0552 (0.0534-0.0573) |
|  | 2005 | 100 | 2 | 1 | 0.0543 (0.0531-0.0558) |
|  | 4225 | 100 | 2 | 3 | 0.0538 (0.0533-0.0542) |
|  | 8138 | 100 | 3 | 2 | 0.0525 (0.0523-0.0528) |
|  | 15105 | 100 | 3 | 3 | 0.0519 (0.05175-0.052) |
| RF | 1182 | 100 | 2 | 3 | 0.0545 (0.0528-0.056) |
|  | 2005 | 100 | 1 | 2 | 0.0539 (0.0529-0.0552) |
|  | 4225 | 100 | 1 | 3 | 0.5260 (0.5255-0.5264) |
|  | 8138 | 100 | 2 | 2 | 0.0519 (0.0517-0.0522) |
|  | 15105 | 100 | 2 | 2 | 0.0510 (0.0509-0.0511) |
| Boosting | 1182 | 100 | 1 | 2 | 0.0574 (0.0556-0.0594) |
|  | 2005 | 100 | 1 | 3 | 0.0563 (0.0553-0.0578) |
|  | 4225 | 100 | 3 | 3 | 0.0552 (0.0544-0.0553) |
|  | 8138 | 100 | 2 | 2 | 0.0550 (0.0545-0.0556) |
|  | 15105 | 100 | 1 | 3 | 0.0548 (0.0546-0.0551) |

**Table S5.** Hyperparameter values used for each scenario when analysis model is Random Forest.

|  |  | **Hyperparameter values** | | |  |
| --- | --- | --- | --- | --- | --- |
| **DGM** | **Sample Size** | **Ntrees** | **Mtry** | **Nodesize** | **Median Brier Score (IQR)** |
| LR-ME | 1182 | 100 | 3 | 2 | 0.0503 (0.0481-0.0526) |
|  | 2005 | 100 | 3 | 2 | 0.0497 (0.0486-0.0510) |
|  | 4225 | 100 | 2 | 3 | 0.0490 (0.0480-0.0504) |
|  | 8138 | 100 | 2 | 2 | 0.0485 (0.0478-0.0493) |
|  | 15105 | 100 | 3 | 3 | 0.0487 (0.0485-0.0489) |
| LR-Neutral | 1182 | 100 | 3 | 3 | 0.0490 (0.0469-0.0514) |
|  | 2005 | 100 | 2 | 2 | 0.0518 (0.0503-0.0537) |
|  | 4225 | 100 | 3 | 3 | 0.0491 (0.0483-0.0492) |
|  | 8138 | 100 | 2 | 4 | 0.0493 (0.0488-0.0499) |
|  | 15105 | 100 | 3 | 2 | 0.0493 (0.0491-0.0496) |
| Bagging | 1182 | 100 | 2 | 2 | 0.0513 (0.0491-0.0531) |
|  | 2005 | 100 | 2 | 3 | 0.0510 (0.0494-0.0523) |
|  | 4225 | 100 | 3 | 3 | 0.0509 (0.0505-0.0513) |
|  | 8138 | 100 | 3 | 2 | 0.0498 (0.0496-0.0501) |
|  | 15105 | 100 | 3 | 3 | 0.0498 (0.0497-0.0499) |
| RF | 1182 | 100 | 2 | 3 | 0.0516 (0.0499-0.0531) |
|  | 2005 | 100 | 2 | 2 | 0.0505 (0.0495-0.0518) |
|  | 4225 | 100 | 3 | 3 | 0.0498 (0.0493-0.0502) |
|  | 8138 | 100 | 3 | 2 | 0.0496 (0.0494-0.0499) |
|  | 15105 | 100 | 2 | 2 | 0.0490 (0.0488-0.0491) |
| Boosting | 1182 | 100 | 3 | 2 | 0.0525 (0.0507-0.0545) |
|  | 2005 | 100 | 2 | 3 | 0.0520 (0.0511-0.0535) |
|  | 4225 | 100 | 2 | 3 | 0.0518 (0.0510-0.0529) |
|  | 8138 | 100 | 2 | 4 | 0.0515 (0.0510-0.0521) |
|  | 15105 | 100 | 2 | 3 | 0.0515 (0.0513-0.0518) |

**Table S6.** Hyperparameter values used for each scenario when analysis model is Boosting.

|  |  | **Hyperparameter values** | | | |  |
| --- | --- | --- | --- | --- | --- | --- |
| **DGM** | **Sample Size** | **n.trees** | **interaction.depth** | **shrinkage** | **n.minobsinnode** | **Median Brier Score (IQR)** |
| LR-ME | 1182 | 100 | 1 | 0.1 | 3 | 0.0496 (0.0478-0.0517) |
|  | 2005 | 100 | 1 | 0.1 | 5 | 0.0481 (0.0469-0.0496) |
|  | 4225 | 100 | 1 | 0.1 | 3 | 0.0473 (0.0469-0.0477) |
|  | 8138 | 100 | 1 | 0.05 | 3 | 0.0475 (0.0473-0.0478) |
|  | 15105 | 100 | 1 | 0.05 | 5 | 0.0470 (0.0468-0.0471) |
| LR-Neutral | 1182 | 100 | 1 | 0.1 | 10 | 0.0501 (0.0484-0.0516) |
|  | 2005 | 100 | 1 | 0.1 | 5 | 0.0496 (0.0486-0.0509) |
|  | 4225 | 100 | 1 | 0.1 | 3 | 0.0482 (0.0475-0.0486) |
|  | 8138 | 100 | 1 | 0.1 | 3 | 0.0479 (0.0477-0.0482) |
|  | 15105 | 100 | 1 | 0.05 | 5 | 0.0475 (0.0474-0.0476) |
| Bagging | 1182 | 100 | 1 | 0.1 | 3 | 0.0515 (0.0497-0.0535) |
|  | 2005 | 100 | 1 | 0.1 | 5 | 0.0502 (0.0492-0.0521) |
|  | 4225 | 100 | 1 | 0.1 | 3 | 0.0495 (0.0487-0.0499) |
|  | 8138 | 100 | 1 | 0.1 | 10 | 0.0491 (0.0488-0.0495) |
|  | 15105 | 100 | 1 | 0.1 | 5 | 0.0510 (0.0509-0.0512) |
| RF | 1182 | 100 | 1 | 0.05 | 3 | 0.0510 (0.0493-0.0525) |
|  | 2005 | 100 | 1 | 0.1 | 5 | 0.0500 (0.0490-0.0513) |
|  | 4225 | 100 | 1 | 0.1 | 3 | 0.0491 (0.0486-0.0495) |
|  | 8138 | 100 | 1 | 0.1 | 3 | 0.0489 (0.0487-0.0492) |
|  | 15105 | 100 | 1 | 0.1 | 5 | 0.0485 (0.0483-0.0486) |
| Boosting | 1182 | 100 | 1 | 0.1 | 10 | 0.0511 (0.0490-0.0546) |
|  | 2005 | 100 | 1 | 0.1 | 10 | 0.0501 (0.0485-0.0512) |
|  | 4225 | 100 | 1 | 0.05 | 3 | 0.0495 (0.0487-0.0496) |
|  | 8138 | 100 | 1 | 0.1 | 5 | 0.0495 (0.0493-0.0497) |
|  | 15105 | 100 | 1 | 0.1 | 5 | 0.0490 (0.0490-0.0491) |

**Heart Failure Dataset**

**Table S7**. Hyperparameter values used for each scenario when analysis model is Bagging.

|  |  | **Hyperparameter values** | | |  |
| --- | --- | --- | --- | --- | --- |
| **DGM** | **Sample Size** | **Nbagg** | **Maxdepth** | **Nodesize** | **Median Brier Score (IQR)** |
| LR-NL | 2922 | 100 | 2 | 3 | 0.0907 (0.0889-0.0927) |
|  | 6157 | 100 | 3 | 3 | 0.0881 (0.0870-0.0895) |
|  | 10448 | 100 | 4 | 2 | 0.0885 (0.0880-0.0889) |
| Bagging | 2922 | 100 | 2 | 2 | 0.0893 (0.0871-0.0917) |
|  | 6157 | 100 | 2 | 2 | 0.0876 (0.0864-0.0891) |
|  | 10448 | 100 | 3 | 1 | 0.0842 (0.0840-0.0845) |
| RF | 2922 | 100 | 1 | 3 | 0.0881 (0.0864-0.0896) |
|  | 6157 | 100 | 2 | 2 | 0.0867 (0.0857-0.0880) |
|  | 10448 | 100 | 2 | 2 | 0.0854 (0.0850-0.0860) |
| Boosting | 2922 | 100 | 3 | 4 | 0.0972 (0.0954-0.0998) |
|  | 6157 | 100 | 2 | 3 | 0.0964 (0.0951-0.0976) |
|  | 10448 | 100 | 2 | 2 | 0.0970 (0.0967-0.0974) |

**Table S8.** Hyperparameter values used for each scenario when analysis model is Random Forest.

|  |  | **Hyperparameter values** | | |  |
| --- | --- | --- | --- | --- | --- |
| **DGM** | **Sample Size** | **Ntrees** | **Mtry** | **Nodesize** | **Median Brier Score (IQR)** |
| LR-NL | 2922 | 100 | 2 | 2 | 0.0942 (0.0924-0.0962) |
|  | 6157 | 100 | 3 | 2 | 0.0891 (0.0881-0.0906) |
|  | 10448 | 100 | 2 | 2 | 0.0876 (0.0872-0.0879) |
| Bagging | 2922 | 100 | 2 | 3 | 0.0870 (0.0846-0.0888) |
|  | 6157 | 100 | 2 | 3 | 0.0856 (0.0844-0.0869) |
|  | 10448 | 100 | 2 | 4 | 0.0813 (0.0809-0.0815) |
| RF | 2922 | 100 | 3 | 2 | 0.0843 (0.0819-0.0865) |
|  | 6157 | 100 | 2 | 4 | 0.0835 (0.0828-0.0850) |
|  | 10448 | 100 | 2 | 2 | 0.0831 (0.0828-0.0834) |
| Boosting | 2922 | 100 | 2 | 3 | 0.0953 (0.0935-0.0973) |
|  | 6157 | 100 | 2 | 2 | 0.0916 (0.0907-0.0930) |
|  | 10448 | 100 | 3 | 4 | 0.0935 (0.0931-0.0938) |

**Table S9.** Hyperparameter values used for each scenario when analysis model is Boosting.

|  |  | **Hyperparameter values** | | |  |  |
| --- | --- | --- | --- | --- | --- | --- |
| **DGM** | **Sample Size** | **n.trees** | **interaction. Depth** | **shrinkage** | **n.minobsinnode** | **Median Brier Score (IQR)** |
| LR-NL | 2922 | 100 | 3 | 0.05 | 3 | 0.0826 (0.0806-0.0848) |
|  | 6157 | 100 | 3 | 0.05 | 5 | 0.0839 (0.0830-0.0855) |
|  | 10448 | 100 | 1 | 0.1 | 5 | 0.0810 (0.0806-0.0815) |
| Bagging | 2922 | 100 | 3 | 0.1 | 5 | 0.0863 (0.0848-0.0885) |
|  | 6157 | 100 | 5 | 0.05 | 3 | 0.0867 (0.0852-0.0883) |
|  | 10448 | 100 | 3 | 0.05 | 5 | 0.0853 (0.0849-0.0858) |
| RF | 2922 | 100 | 1 | 0.1 | 10 | 0.0835 (0.0811-0.0853) |
|  | 6157 | 100 | 1 | 0.1 | 5 | 0.0831 (0.0819-0.0844) |
|  | 10448 | 100 | 3 | 0.05 | 5 | 0.0834 (0.0829-0.0839) |
| Boosting | 2922 | 100 | 3 | 0.1 | 5 | 0.0896 (0.0876-0.0918) |
|  | 6157 | 100 | 3 | 0.1 | 3 | 0.0865 (0.0856-0.0881) |
|  | 10448 | 100 | 2 | 0.05 | 5 | 0.0880 (0.0876-0.0885) |

**3. Summary of performance measures when class imbalance techniques were used**

The ML based analysis models were fitted using a variety of approaches for dealing with class imbalance problem:

1. Resampling techniques (down sampling, up sampling, ROSE, SMOTE),
2. Calibration correction using Platt scaling (CC-Platt) and isotonic regression (CC-IsoReg)
3. Calibration correction with resampling techniques (CC- Platt w/ SMOTE and CC-IsoReg w/ SMOTE)
4. Cost-sensitive learning (CSL) with ratio of class weights between the majority and minority classes as 1:10 and 1:100.

These models were applied to 10 randomly generated simulated datasets under different data-generation models for both datasets. We used the smallest sample size scenarios for each dataset that takes the least time to train the model.

**RESULTS: MINAP Dataset**

**Table S10**. Summary (median) of performance measures when data-generation model is logistic regression with main effects (Bold values indicate the best performing strategy in each ML model for each performance metric).

| **Analysis Model** | **MAPE** | **Brier Score** | **C-Statistic** | **Calibration in the large** | **Calibration**  **slope** |
| --- | --- | --- | --- | --- | --- |
| LR-ME | 0.016 | 0.047 | 0.851 | -0.0001 | 0.884 |
| Bagg-original | 0.049 | **0.053** | 0.749 | -0.0090 | 0.688 |
| Bagg-down | 0.299 | 0.180 | **0.793** | -0.2974 | 0.783 |
| Bagg-up | 0.070 | 0.065 | 0.699 | -0.0232 | 0.454 |
| Bagg-ROSE | 0.099 | 0.083 | 0.762 | -0.0777 | 0.490 |
| Bagg-SMOTE | 0.076 | 0.068 | 0.723 | -0.0400 | 0.477 |
| Bagg-CC-Platt | **0.046** | 0.054 | 0.784 | **-0.0003** | **0.982** |
| Bagg-CC-IsoReg | 0.047 | 0.055 | 0.687 | 0.0246 | 0.850 |
| Bagg-CC-Platt w/ SMOTE | 0.048 | 0.057 | 0.771 | 0.0102 | 0.670 |
| Bagg-CC-IsoReg w/ SMOTE | 0.066 | 0.067 | 0.656 | -0.0088 | 0.537 |
| Bagg-CSL-weight0.1 | 0.049 | 0.054 | 0.749 | -0.0092 | 0.686 |
| Bagg-CSL-weight0.01 | 0.049 | **0.053** | 0.748 | -0.0087 | 0.684 |
| RF-original | **0.036** | **0.050** | 0.778 | 0.0122 | 0.861 |
| RF-down | 0.301 | 0.170 | 0.814 | -0.3003 | **1.009** |
| RF-up | 0.095 | 0.067 | 0.801 | -0.0864 | 0.911 |
| RF-ROSE | 0.127 | 0.086 | **0.821** | -0.1251 | 0.782 |
| RF-SMOTE | 0.068 | 0.060 | 0.792 | -0.0522 | 0.776 |
| RF-CC-Platt | 0.042 | 0.052 | 0.803 | **-0.0019** | 0.994 |
| RF-CC-IsoReg | 0.047 | 0.056 | 0.716 | -0.0027 | 0.775 |
| RF-CC-Platt w/ SMOTE | 0.041 | 0.052 | 0.809 | 0.0027 | 1.029 |
| RF-CC-IsoReg w/ SMOTE | 0.049 | 0.056 | 0.723 | -0.0025 | 0.863 |
| RF-CSL-weight0.1 | **0.036** | **0.050** | 0.779 | 0.0117 | 0.855 |
| RF-CSL-weight0.01 | **0.036** | **0.050** | 0.779 | 0.0116 | 0.865 |
| Boost-original | **0.028** | **0.049** | **0.837** | **-0.0004** | 0.916 |
| Boost-RS-down | 0.290 | 0.174 | 0.815 | -0.2894 | 0.818 |
| Boost-RS-up | 0.255 | 0.139 | 0.831 | -0.2549 | 1.068 |
| Boost-RS-ROSE | 0.170 | 0.087 | 0.836 | -0.1697 | 1.133 |
| Boost-RS-SMOTE | 0.175 | 0.099 | 0.823 | -0.1743 | **0.966** |
| Boost-CC-Platt | 0.047 | 0.054 | 0.785 | -0.0007 | 0.952 |
| Boost-CC-IsoReg | 0.047 | 0.055 | 0.685 | 0.0237 | 0.817 |
| Boost-CC-Platt w/ SMOTE | 0.049 | 0.056 | 0.769 | 0.0061 | 0.891 |
| Boost-CC-IsoReg w/ SMOTE | 0.063 | 0.066 | 0.659 | -0.0052 | 0.550 |
| Boost-CSL-weight0.1 | 0.054 | 0.057 | 0.820 | 0.0533 | 0.825 |
| Boost-CSL-weight0.01 | 0.059 | 0.060 | 0.808 | 0.0593 | 0.781 |

**Table S11.** Summary (mean) of confusion matrix when data-generation model is logistic regression with main effects.

|  | **Classification Threshold=0.50** | | | | |  | **Classification Threshold=0.063** | | | | |
| --- | --- | --- | --- | --- | --- | --- | --- | --- | --- | --- | --- |
| **Analysis Model** | **Accuracy** | **TP** | **TN** | **FP** | **FN** |  | **Accuracy** | **TP** | **TN** | **FP** | **FN** |
| LR-ME | 0.942 | 2880 | 201795 | 1783 | 10758 |  | 0.799 | 10040 | 163460 | 40118 | 3598 |
| Bagg-original | 0.937 | 2566 | 200857 | 2721 | 11072 |  | 0.744 | 9428 | 152139 | 51439 | 4210 |
| Bagg-down | 0.738 | 9730 | 150551 | 53027 | 3908 |  | 0.194 | 13369 | 28864 | 174714 | 269 |
| Bagg-up | 0.920 | 2651 | 197173 | 6406 | 10986 |  | 0.729 | 9076 | 149326 | 54252 | 4562 |
| Bagg-ROSE | 0.891 | 6419 | 187061 | 16517 | 7219 |  | 0.687 | 10597 | 138627 | 64951 | 3041 |
| Bagg-SMOTE | 0.915 | 3621 | 195192 | 8387 | 10017 |  | 0.698 | 9787 | 141913 | 61665 | 3851 |
| Bagg-CC-Platt | 0.937 | 1803 | 201749 | 1829 | 11835 |  | 0.802 | 7863 | 166384 | 37194 | 5774 |
| Bagg-CC-IsoReg | 0.936 | 723 | 203052 | 496 | 12945 |  | 0.857 | 5853 | 180359 | 23188 | 7816 |
| Bagg-CC-Platt w/ SMOTE | 0.931 | 1749 | 200529 | 3012 | 11926 |  | 0.853 | 6380 | 178797 | 24743 | 7296 |
| Bagg-CC-IsoReg w/ SMOTE | 0.928 | 2337 | 199153 | 4395 | 11331 |  | 0.803 | 7006 | 167481 | 36066 | 6662 |
| Bagg-CSL-weight0.1 | 0.936 | 2543 | 200865 | 2713 | 11094 |  | 0.742 | 9393 | 151842 | 51736 | 4245 |
| Bagg-CSL-weight0.01 | 0.936 | 2545 | 200858 | 2720 | 11092 |  | 0.745 | 9415 | 152401 | 51178 | 4223 |
| RF-original | 0.940 | 1254 | 202932 | 647 | 12384 |  | 0.809 | 8864 | 166808 | 36770 | 4774 |
| RF-down | 0.753 | 9934 | 153624 | 49954 | 3704 |  | 0.134 | 13521 | 15604 | 187974 | 117 |
| RF-up | 0.921 | 3948 | 196093 | 7486 | 9690 |  | 0.490 | 12401 | 93990 | 109588 | 1236 |
| RF-ROSE | 0.883 | 7159 | 184620 | 18958 | 6479 |  | 0.492 | 12417 | 94529 | 109049 | 1221 |
| RF-SMOTE | 0.927 | 3858 | 197542 | 6036 | 9780 |  | 0.612 | 11493 | 121490 | 82088 | 2145 |
| RF-CC-Platt | 0.938 | 2254 | 201548 | 2030 | 11384 |  | 0.801 | 8352 | 165712 | 37866 | 5286 |
| RF-CC-IsoReg | 0.935 | 2858 | 200169 | 3390 | 10800 |  | 0.860 | 7070 | 179695 | 23864 | 6588 |
| RF-CC-Platt w/ SMOTE | 0.937 | 1383 | 202167 | 1399 | 12267 |  | 0.811 | 8534 | 167670 | 35895 | 5117 |
| RF-CC-IsoReg w/ SMOTE | 0.937 | 1436 | 202091 | 1457 | 12232 |  | 0.778 | 8159 | 160744 | 42803 | 5510 |
| RF-CSL-weight0.1 | 0.940 | 1332 | 202877 | 701 | 12306 |  | 0.808 | 8936 | 166590 | 36988 | 4702 |
| RF-CSL-weight0.01 | 0.940 | 1293 | 202898 | 680 | 12345 |  | 0.805 | 8942 | 165869 | 37709 | 4696 |
| Boost-original | 0.941 | 2519 | 201817 | 1761 | 11119 |  | 0.802 | 9563 | 164722 | 38856 | 4075 |
| Boost-RS-down | 0.749 | 9971 | 152770 | 50808 | 3667 |  | 0.155 | 13514 | 20250 | 183328 | 124 |
| Boost-RS-up | 0.808 | 9331 | 166073 | 37505 | 4307 |  | 0.136 | 13554 | 15974 | 187604 | 84 |
| Boost-RS-ROSE | 0.896 | 6740 | 187922 | 15665 | 6890 |  | 0.125 | 13565 | 13519 | 190068 | 65 |
| Boost-RS-SMOTE | 0.866 | 7623 | 180424 | 23154 | 6015 |  | 0.252 | 13281 | 41519 | 162059 | 357 |
| Boost-CC-Platt | 0.937 | 1829 | 201650 | 1928 | 11809 |  | 0.813 | 7805 | 168830 | 34748 | 5833 |
| Boost-CC-IsoReg | 0.938 | 795 | 203014 | 533 | 12874 |  | 0.856 | 5889 | 180054 | 23494 | 7780 |
| Boost-CC-Platt w/ SMOTE | 0.934 | 1127 | 201762 | 1772 | 12556 |  | 0.825 | 6958 | 172208 | 31326 | 6725 |
| Boost-CC-IsoReg w/ SMOTE | 0.929 | 2248 | 199528 | 4020 | 11420 |  | 0.814 | 6890 | 169981 | 33566 | 6779 |
| Boost-CSL-weight0.1 | 0.939 | 430 | 203465 | 113 | 13208 |  | 0.939 | 2680 | 201178 | 2400 | 10957 |
| Boost-CSL-weight0.01 | 0.938 | 121 | 203555 | 23 | 13517 |  | 0.940 | 1397 | 202821 | 758 | 12241 |

**Table S12.** Summary (median) of performance measures when data-generation model is neutral (Bold values indicate the best performing strategy in each ML model for each performance metric).

| **Analysis Model** | **MAPE** | **Brier Score** | **C-Statistic** | **Calibration in the large** | **Calibration slope** |
| --- | --- | --- | --- | --- | --- |
| LR-ME | 0.028 | 0.050 | 0.850 | 0.0025 | 0.810 |
| Bagg-original | 0.048 | **0.054** | 0.733 | -0.0057 | 0.620 |
| Bagg-down | 0.285 | 0.180 | **0.818** | -0.2843 | 0.667 |
| Bagg-up | 0.066 | 0.063 | 0.782 | -0.0218 | 0.217 |
| Bagg-ROSE | 0.499 | 0.488 | 0.678 | -0.4909 | 0.158 |
| Bagg-SMOTE | 0.074 | 0.068 | 0.790 | -0.0365 | 0.228 |
| Bagg-CC-Platt | 0.051 | **0.054** | 0.803 | 0.0044 | 1.267 |
| Bagg-CC-IsoReg | **0.047** | 0.055 | 0.684 | 0.0272 | 0.825 |
| Bagg-CC-Platt w/ SMOTE | 0.052 | 0.055 | 0.787 | 0.0043 | **1.012** |
| Bagg-CC-IsoReg w/ SMOTE | 0.059 | 0.063 | 0.667 | **0.0026** | 0.576 |
| Bagg-CSL-weight0.1 | 0.048 | **0.054** | 0.733 | -0.0048 | 0.626 |
| Bagg-CSL-weight0.01 | 0.048 | **0.054** | 0.734 | -0.0056 | 0.629 |
| RF-original | **0.035** | **0.050** | 0.775 | 0.0137 | 0.850 |
| RF-down | 0.280 | 0.159 | **0.838** | -0.2802 | 1.008 |
| RF-up | 0.086 | 0.065 | 0.835 | -0.0764 | 0.777 |
| RF-ROSE | 0.384 | 0.275 | 0.834 | -0.3830 | 0.953 |
| RF-SMOTE | 0.065 | 0.060 | 0.836 | -0.0511 | 0.588 |
| RF-CC-Platt | 0.046 | 0.053 | 0.820 | 0.0056 | 1.194 |
| RF-CC-IsoReg | 0.045 | 0.054 | 0.763 | 0.0045 | 0.930 |
| RF-CC-Platt w/ SMOTE | 0.044 | 0.053 | 0.833 | 0.0032 | **0.998** |
| RF-CC-IsoReg w/ SMOTE | 0.046 | 0.055 | 0.733 | **0.0027** | 0.860 |
| RF-CSL-weight0.1 | **0.036** | **0.050** | 0.777 | 0.0129 | 0.855 |
| RF-CSL-weight0.01 | **0.036** | **0.050** | 0.776 | 0.0129 | 0.851 |
| Boost-original | **0.031** | **0.050** | **0.848** | **0.0026** | 0.913 |
| Boost-RS-down | 0.271 | 0.165 | 0.835 | -0.2707 | 0.773 |
| Boost-RS-up | 0.244 | 0.135 | 0.841 | -0.2441 | **1.012** |
| Boost-RS-ROSE | 0.525 | 0.471 | 0.687 | -0.5242 | 0.621 |
| Boost-RS-SMOTE | 0.074 | 0.068 | 0.790 | -0.0365 | 0.228 |
| Boost-CC-Platt | 0.051 | 0.054 | 0.803 | 0.0040 | 1.333 |
| Boost-CC-IsoReg | 0.047 | 0.055 | 0.683 | 0.0276 | 0.825 |
| Boost-CC-Platt w/ SMOTE | 0.052 | 0.055 | 0.788 | 0.0052 | 1.025 |
| Boost-CC-IsoReg w/ SMOTE | 0.059 | 0.063 | 0.663 | 0.0027 | 0.557 |
| Boost-CSL-weight0.1 | 0.056 | 0.059 | 0.836 | 0.0557 | 0.911 |
| Boost-CSL-weight0.01 | 0.060 | 0.061 | 0.824 | 0.0603 | 0.863 |

**Table S13.** Summary (mean) of confusion matrix when data-generation model is neutral.

|  | **Classification Threshold=0.50** | | | | |  | **Classification Threshold=0.063** | | | | |
| --- | --- | --- | --- | --- | --- | --- | --- | --- | --- | --- | --- |
| **Analysis Model** | **Accuracy** | **TP** | **TN** | **FP** | **FN** |  | **Accuracy** | **TP** | **TN** | **FP** | **FN** |
| LR-ME | 0.938 | 2076 | 201728 | 1851 | 11561 |  | 0.802 | 10062 | 164125 | 39454 | 3575 |
| Bagg-original | 0.934 | 2023 | 200854 | 2725 | 11615 |  | 0.760 | 9733 | 155314 | 48264 | 3904 |
| Bagg-down | 0.730 | 10381 | 148094 | 55484 | 3256 |  | 0.274 | 13372 | 46185 | 157394 | 266 |
| Bagg-up | 0.920 | 2762 | 197149 | 6429 | 10875 |  | 0.741 | 9418 | 151504 | 52075 | 4219 |
| Bagg-ROSE | 0.492 | 10492 | 96395 | 107184 | 3146 |  | 0.395 | 12429 | 73423 | 130156 | 1208 |
| Bagg-SMOTE | 0.913 | 3608 | 194664 | 8915 | 10030 |  | 0.722 | 9978 | 146751 | 56828 | 3660 |
| Bagg-CC-Platt | 0.936 | 904 | 202491 | 1088 | 12734 |  | 0.839 | 7433 | 174788 | 28791 | 6204 |
| Bagg-CC-IsoReg | 0.937 | 554 | 202961 | 618 | 13083 |  | 0.837 | 7396 | 174420 | 29159 | 6241 |
| Bagg-CC-Platt w/ SMOTE | 0.936 | 736 | 202520 | 1059 | 12901 |  | 0.832 | 7240 | 173455 | 30123 | 6397 |
| Bagg-CC-IsoReg w/ SMOTE | 0.929 | 1687 | 200024 | 3555 | 11951 |  | 0.803 | 8179 | 166254 | 37325 | 5459 |
| Bagg-CSL-weight0.1 | 0.935 | 1950 | 201039 | 2540 | 11688 |  | 0.761 | 9742 | 155636 | 47943 | 3896 |
| Bagg-CSL-weight0.01 | 0.934 | 2004 | 200952 | 2626 | 11634 |  | 0.758 | 9785 | 154869 | 48709 | 3853 |
| RF-original | 0.938 | 863 | 202978 | 600 | 12775 |  | 0.805 | 9366 | 165479 | 38100 | 4271 |
| RF-down | 0.769 | 10277 | 156781 | 46798 | 3361 |  | 0.160 | 13552 | 21183 | 182395 | 86 |
| RF-up | 0.920 | 3898 | 196037 | 7542 | 9740 |  | 0.545 | 12578 | 105710 | 97868 | 1060 |
| RF-ROSE | 0.506 | 10833 | 99123 | 104456 | 2804 |  | 0.283 | 13268 | 48224 | 155354 | 369 |
| RF-SMOTE | 0.923 | 4026 | 196378 | 7201 | 9611 |  | 0.647 | 11804 | 128810 | 74769 | 1834 |
| RF-CC-Platt | 0.938 | 1358 | 202315 | 1263 | 12280 |  | 0.850 | 7723 | 176814 | 26764 | 5915 |
| RF-CC-IsoReg | 0.937 | 1248 | 202243 | 1335 | 12390 |  | 0.760 | 9741 | 155439 | 48140 | 3896 |
| RF-CC-Platt w/ SMOTE | 0.936 | 1327 | 201979 | 1600 | 12310 |  | 0.828 | 8647 | 171306 | 32273 | 4990 |
| RF-CC-IsoReg w/ SMOTE | 0.935 | 1161 | 201958 | 1621 | 12476 |  | 0.762 | 9869 | 155725 | 47854 | 3769 |
| RF-CSL-weight0.1 | 0.938 | 893 | 202946 | 632 | 12744 |  | 0.803 | 9446 | 165003 | 38576 | 4192 |
| RF-CSL-weight0.01 | 0.938 | 865 | 202977 | 602 | 12772 |  | 0.803 | 9454 | 164885 | 38694 | 4183 |
| Boost-original | 0.938 | 2025 | 201818 | 1761 | 11612 |  | 0.808 | 9695 | 165893 | 37685 | 3942 |
| Boost-RS-down | 0.766 | 10156 | 156299 | 47279 | 3482 |  | 0.196 | 13520 | 29059 | 174520 | 117 |
| Boost-RS-up | 0.812 | 9770 | 166607 | 36972 | 3868 |  | 0.153 | 13560 | 19676 | 183902 | 77 |
| Boost-RS-ROSE | 0.501 | 10502 | 98284 | 105295 | 3136 |  | 0.118 | 13470 | 12160 | 191418 | 168 |
| Boost-RS-SMOTE | 0.869 | 7701 | 181116 | 22463 | 5936 |  | 0.325 | 13264 | 57228 | 146350 | 373 |
| Boost-CC-Platt | 0.936 | 1045 | 202207 | 1371 | 12592 |  | 0.842 | 7226 | 175619 | 27959 | 6411 |
| Boost-CC-IsoReg | 0.937 | 535 | 202930 | 648 | 13103 |  | 0.838 | 7324 | 174738 | 28841 | 6313 |
| Boost-CC-Platt w/ SMOTE | 0.936 | 629 | 202765 | 813 | 13009 |  | 0.833 | 7291 | 173563 | 30016 | 6346 |
| Boost-CC-IsoReg w/ SMOTE | 0.929 | 1692 | 200059 | 3520 | 11946 |  | 0.805 | 8200 | 166553 | 37025 | 5438 |
| Boost-CSL-weight0.1 | 0.938 | 166 | 203506 | 73 | 13471 |  | 0.938 | 1930 | 201808 | 1771 | 11707 |
| Boost-CSL-weight0.01 | 0.937 | 37 | 203564 | 15 | 13601 |  | 0.939 | 818 | 203052 | 526 | 12820 |

**Table S14.** Summary (median) of performance measures when data-generation model is bagging (Bold values indicate the best performing strategy in each ML model for each performance metric).

| **Analysis Model** | **MAPE** | **Brier Score** | **C-Statistic** | **Calibration in the large** | **Calibration slope** |
| --- | --- | --- | --- | --- | --- |
| LR-ME | 0.069 | 0.051 | 0.858 | -0.0011 | 0.879 |
| Bagg-original | 0.077 | **0.055** | 0.747 | -0.0067 | 0.645 |
| Bagg-down | 0.291 | 0.179 | 0.817 | -0.2745 | 0.580 |
| Bagg-up | 0.094 | 0.066 | 0.797 | -0.0248 | 0.236 |
| Bagg-ROSE | 0.200 | 0.159 | 0.812 | -0.1657 | 0.367 |
| Bagg-SMOTE | 0.099 | 0.067 | 0.815 | -0.0394 | 0.280 |
| Bagg-CC-Platt | 0.079 | **0.055** | **0.821** | **-0.0013** | 0.942 |
| Bagg-CC-IsoReg | **0.066** | **0.055** | 0.723 | 0.0236 | 0.721 |
| Bagg-CC-Platt w/ SMOTE | 0.084 | 0.057 | 0.811 | -0.0035 | **0.989** |
| Bagg-CC-IsoReg w/ SMOTE | 0.080 | 0.061 | 0.707 | -0.0007 | 0.588 |
| Bagg-CSL-weight0.1 | 0.076 | **0.055** | 0.747 | -0.0069 | 0.646 |
| Bagg-CSL-weight0.01 | 0.077 | **0.055** | 0.746 | -0.0069 | 0.637 |
| RF-original | **0.066** | **0.051** | 0.787 | 0.0112 | 0.876 |
| RF-down | 0.286 | 0.156 | 0.848 | -0.2718 | 1.030 |
| RF-up | 0.127 | 0.067 | 0.845 | -0.0813 | 0.870 |
| RF-ROSE | 0.193 | 0.115 | **0.853** | -0.1655 | 0.850 |
| RF-SMOTE | 0.104 | 0.062 | 0.845 | -0.0536 | 0.646 |
| RF-CC-Platt | 0.077 | 0.053 | 0.836 | **-0.0021** | 1.013 |
| RF-CC-IsoReg | 0.077 | 0.055 | 0.762 | -0.0059 | 0.870 |
| RF-CC-Platt w/ SMOTE | 0.079 | 0.054 | 0.842 | -0.0035 | **1.001** |
| RF-CC-IsoReg w/ SMOTE | 0.078 | 0.056 | 0.766 | -0.0048 | 0.799 |
| RF-CSL-weight0.1 | **0.066** | **0.051** | 0.788 | 0.0108 | 0.877 |
| RF-CSL-weight0.01 | **0.066** | **0.051** | 0.788 | 0.0108 | 0.873 |
| Boost-original | 0.070 | **0.051** | **0.857** | **0.0005** | 0.910 |
| Boost-RS-down | 0.272 | 0.153 | 0.836 | -0.2561 | 0.815 |
| Boost-RS-up | 0.259 | 0.134 | 0.855 | -0.2429 | **1.012** |
| Boost-RS-ROSE | 0.265 | 0.166 | 0.823 | -0.2395 | 0.991 |
| Boost-RS-SMOTE | 0.099 | 0.067 | 0.815 | -0.0394 | 0.280 |
| Boost-CC-Platt | 0.079 | 0.055 | 0.821 | -0.0019 | 0.961 |
| Boost-CC-IsoReg | 0.066 | 0.055 | 0.723 | 0.0234 | 0.723 |
| Boost-CC-Platt w/ SMOTE | 0.085 | 0.056 | 0.810 | -0.0028 | 1.022 |
| Boost-CC-IsoReg w/ SMOTE | 0.080 | 0.062 | 0.704 | **-0.0005** | 0.576 |
| Boost-CSL-weight0.1 | **0.061** | 0.060 | 0.847 | 0.0560 | 0.885 |
| Boost-CSL-weight0.01 | 0.064 | 0.063 | 0.831 | 0.0622 | 0.861 |

**Table S15.** Summary (mean) of confusion matrix when data-generation model is bagging.

|  | **Classification Threshold=0.50** | | | | |  | **Classification Threshold=0.063** | | | | |
| --- | --- | --- | --- | --- | --- | --- | --- | --- | --- | --- | --- |
| **Analysis Model** | **Accuracy** | **TP** | **TN** | **FP** | **FN** |  | **Accuracy** | **TP** | **TN** | **FP** | **FN** |
| LR-ME | 0.937 | 2528 | 200910 | 2090 | 11688 |  | 0.784 | 11003 | 159319 | 43681 | 3212 |
| Bagg-original | 0.931 | 2579 | 199730 | 3270 | 11637 |  | 0.761 | 10565 | 154804 | 48197 | 3651 |
| Bagg-down | 0.735 | 10682 | 148945 | 54056 | 3534 |  | 0.303 | 13958 | 51767 | 151233 | 258 |
| Bagg-up | 0.915 | 3022 | 195696 | 7305 | 11194 |  | 0.736 | 10392 | 149562 | 53438 | 3824 |
| Bagg-ROSE | 0.817 | 7536 | 169950 | 33050 | 6680 |  | 0.588 | 12655 | 115137 | 87863 | 1561 |
| Bagg-SMOTE | 0.912 | 4027 | 194138 | 8862 | 10189 |  | 0.709 | 11175 | 142802 | 60199 | 3041 |
| Bagg-CC-Platt | 0.933 | 2000 | 200731 | 2269 | 12216 |  | 0.831 | 8693 | 171754 | 31246 | 5523 |
| Bagg-CC-IsoReg | 0.935 | 757 | 202266 | 735 | 13458 |  | 0.803 | 9173 | 165182 | 37819 | 5043 |
| Bagg-CC-Platt w/ SMOTE | 0.931 | 1371 | 200930 | 2070 | 12845 |  | 0.811 | 8742 | 167491 | 35509 | 5474 |
| Bagg-CC-IsoReg w/ SMOTE | 0.928 | 1637 | 199981 | 3019 | 12579 |  | 0.745 | 10108 | 151694 | 51306 | 4108 |
| Bagg-CSL-weight0.1 | 0.931 | 2643 | 199666 | 3335 | 11573 |  | 0.761 | 10607 | 154774 | 48227 | 3609 |
| Bagg-CSL-weight0.01 | 0.931 | 2594 | 199662 | 3339 | 11621 |  | 0.761 | 10569 | 154734 | 48266 | 3646 |
| RF-original | 0.936 | 1265 | 202134 | 866 | 12951 |  | 0.795 | 10372 | 162249 | 40752 | 3844 |
| RF-down | 0.772 | 10885 | 156707 | 46293 | 3331 |  | 0.192 | 14127 | 27498 | 175503 | 89 |
| RF-up | 0.916 | 4393 | 194575 | 8425 | 9823 |  | 0.530 | 13388 | 101802 | 101199 | 828 |
| RF-ROSE | 0.819 | 8467 | 169507 | 33493 | 5749 |  | 0.479 | 13521 | 90441 | 112559 | 694 |
| RF-SMOTE | 0.919 | 4284 | 195434 | 7566 | 9931 |  | 0.639 | 12644 | 126193 | 76807 | 1572 |
| RF-CC-Platt | 0.935 | 2283 | 200859 | 2142 | 11933 |  | 0.836 | 9143 | 172350 | 30650 | 5073 |
| RF-CC-IsoReg | 0.935 | 2170 | 200851 | 2150 | 12046 |  | 0.706 | 11259 | 141994 | 61006 | 2957 |
| RF-CC-Platt w/ SMOTE | 0.933 | 1672 | 200893 | 2108 | 12544 |  | 0.802 | 9993 | 164279 | 38721 | 4222 |
| RF-CC-IsoReg w/ SMOTE | 0.932 | 1298 | 201200 | 1801 | 12918 |  | 0.693 | 11695 | 138942 | 64058 | 2521 |
| RF-CSL-weight0.1 | 0.936 | 1314 | 202080 | 920 | 12902 |  | 0.794 | 10402 | 161977 | 41023 | 3814 |
| RF-CSL-weight0.01 | 0.936 | 1325 | 202069 | 931 | 12891 |  | 0.795 | 10372 | 162254 | 40746 | 3844 |
| Boost-original | 0.936 | 2439 | 200938 | 2063 | 11777 |  | 0.799 | 10500 | 163042 | 39959 | 3716 |
| Boost-RS-down | 0.785 | 10251 | 160211 | 42789 | 3965 |  | 0.187 | 14114 | 26402 | 176598 | 102 |
| Boost-RS-up | 0.811 | 10185 | 165968 | 37032 | 4031 |  | 0.138 | 14178 | 15840 | 187160 | 38 |
| Boost-RS-ROSE | 0.809 | 8244 | 167547 | 35453 | 5972 |  | 0.104 | 14155 | 8430 | 194570 | 61 |
| Boost-RS-SMOTE | 0.865 | 8106 | 179835 | 23165 | 6110 |  | 0.323 | 13921 | 56136 | 146864 | 295 |
| Boost-CC-Platt | 0.933 | 1991 | 200744 | 2257 | 12225 |  | 0.828 | 8814 | 171101 | 31900 | 5402 |
| Boost-CC-IsoReg | 0.935 | 771 | 202270 | 731 | 13445 |  | 0.803 | 9193 | 165319 | 37681 | 5023 |
| Boost-CC-Platt w/ SMOTE | 0.932 | 1154 | 201243 | 1757 | 13062 |  | 0.807 | 8869 | 166378 | 36622 | 5346 |
| Boost-CC-IsoReg w/ SMOTE | 0.928 | 1622 | 199993 | 3008 | 12594 |  | 0.750 | 10085 | 152770 | 50230 | 4131 |
| Boost-CSL-weight0.1 | 0.935 | 339 | 202843 | 157 | 13877 |  | 0.935 | 2651 | 200508 | 2493 | 11565 |
| Boost-CSL-weight0.01 | 0.935 | 84 | 202972 | 28 | 14132 |  | 0.936 | 1095 | 202326 | 674 | 13121 |

**Table S16.** Summary (median) of performance measures when data-generation model is random forest (Bold values indicate the best performing strategy in each ML model for each performance metric).

| **Analysis Model** | **MAPE** | **Brier Score** | **C-Statistic** | **Calibration in the large** | **Calibration slope** |
| --- | --- | --- | --- | --- | --- |
| LR-ME | 0.065 | 0.050 | 0.861 | -0.0010 | 0.862 |
| Bagg-original | 0.074 | **0.055** | 0.741 | -0.0091 | 0.627 |
| Bagg-down | 0.289 | 0.170 | **0.830** | -0.2751 | 0.747 |
| Bagg-up | 0.092 | 0.066 | 0.794 | -0.0250 | 0.230 |
| Bagg-ROSE | 0.272 | 0.242 | 0.779 | -0.2422 | 0.270 |
| Bagg-SMOTE | 0.097 | 0.068 | 0.810 | -0.0388 | 0.256 |
| Bagg-CC-Platt | 0.076 | 0.056 | 0.821 | -0.0019 | 0.969 |
| Bagg-CC-IsoReg | **0.065** | **0.055** | 0.715 | 0.0232 | 0.740 |
| Bagg-CC-Platt w/ SMOTE | 0.082 | 0.057 | 0.802 | -0.0028 | **0.950** |
| Bagg-CC-IsoReg w/ SMOTE | 0.080 | 0.063 | 0.702 | **-0.0006** | 0.585 |
| Bagg-CSL-weight0.1 | 0.076 | 0.055 | 0.741 | -0.0093 | 0.631 |
| Bagg-CSL-weight0.01 | 0.075 | **0.055** | 0.739 | -0.0091 | 0.629 |
| RF-original | **0.062** | **0.051** | 0.789 | 0.0111 | 0.872 |
| RF-down | 0.292 | 0.159 | 0.845 | -0.2784 | 1.032 |
| RF-up | 0.123 | 0.066 | 0.845 | -0.0793 | 0.845 |
| RF-ROSE | 0.248 | 0.163 | **0.849** | -0.2252 | 0.851 |
| RF-SMOTE | 0.101 | 0.062 | 0.844 | -0.0525 | 0.630 |
| RF-CC-Platt | 0.072 | 0.054 | 0.837 | -0.0022 | 0.929 |
| RF-CC-IsoReg | 0.071 | 0.055 | 0.784 | **-0.0011** | 0.774 |
| RF-CC-Platt w/ SMOTE | 0.075 | 0.054 | 0.839 | -0.0015 | **0.979** |
| RF-CC-IsoReg w/ SMOTE | 0.076 | 0.056 | 0.756 | -0.0032 | 0.804 |
| RF-CSL-weight0.1 | **0.063** | **0.051** | 0.789 | 0.0117 | 0.875 |
| RF-CSL-weight0.01 | **0.063** | **0.051** | 0.787 | 0.0113 | 0.870 |
| Boost-original | 0.066 | **0.051** | **0.860** | -0.0005 | 0.909 |
| Boost-RS-down | 0.288 | 0.166 | 0.840 | -0.2750 | 0.808 |
| Boost-RS-up | 0.261 | 0.136 | 0.858 | -0.2464 | **1.018** |
| Boost-RS-ROSE | 0.319 | 0.235 | 0.793 | -0.2947 | 0.862 |
| Boost-RS-SMOTE | 0.097 | 0.068 | 0.810 | -0.0388 | 0.256 |
| Boost-CC-Platt | 0.076 | 0.056 | 0.822 | -0.0011 | 0.922 |
| Boost-CC-IsoReg | 0.065 | 0.055 | 0.717 | 0.0235 | 0.742 |
| Boost-CC-Platt w/ SMOTE | 0.082 | 0.057 | 0.802 | -0.0033 | 0.972 |
| Boost-CC-IsoReg w/ SMOTE | 0.079 | 0.063 | 0.701 | **-0.0001** | 0.590 |
| Boost-CSL-weight0.1 | **0.059** | 0.059 | 0.847 | 0.0557 | 0.872 |
| Boost-CSL-weight0.01 | 0.063 | 0.062 | 0.839 | 0.0617 | 0.858 |

**Table S17.** Summary (mean) of confusion matrix when data-generation model is random forest.

|  | **Classification Threshold=0.50** | | | | |  | **Classification Threshold=0.063** | | | | |
| --- | --- | --- | --- | --- | --- | --- | --- | --- | --- | --- | --- |
| **Analysis Model** | **Accuracy** | **TP** | **TN** | **FP** | **FN** |  | **Accuracy** | **TP** | **TN** | **FP** | **FN** |
| LR-ME | 0.937 | 2546 | 200935 | 2236 | 11499 |  | 0.794 | 10843 | 161613 | 41559 | 3201 |
| Bagg-original | 0.931 | 2591 | 199702 | 3470 | 11454 |  | 0.758 | 10505 | 154109 | 49062 | 3540 |
| Bagg-down | 0.746 | 10726 | 151255 | 51917 | 3319 |  | 0.266 | 13869 | 43916 | 159256 | 176 |
| Bagg-up | 0.915 | 2962 | 195756 | 7415 | 11083 |  | 0.738 | 10131 | 150118 | 53053 | 3914 |
| Bagg-ROSE | 0.730 | 8616 | 150055 | 53117 | 5429 |  | 0.573 | 12276 | 112294 | 90878 | 1769 |
| Bagg-SMOTE | 0.913 | 3950 | 194294 | 8877 | 10094 |  | 0.714 | 10846 | 144353 | 58819 | 3199 |
| Bagg-CC-Platt | 0.933 | 1891 | 200696 | 2476 | 12154 |  | 0.831 | 8491 | 171922 | 31249 | 5554 |
| Bagg-CC-IsoReg | 0.935 | 594 | 202519 | 652 | 13451 |  | 0.831 | 8140 | 172321 | 30850 | 5905 |
| Bagg-CC-Platt w/ SMOTE | 0.931 | 1169 | 201082 | 2090 | 12876 |  | 0.811 | 8338 | 167831 | 35341 | 5707 |
| Bagg-CC-IsoReg w/ SMOTE | 0.927 | 1623 | 199741 | 3431 | 12422 |  | 0.788 | 8869 | 162337 | 40835 | 5176 |
| Bagg-CSL-weight0.1 | 0.931 | 2591 | 199641 | 3531 | 11454 |  | 0.757 | 10580 | 153864 | 49307 | 3465 |
| Bagg-CSL-weight0.01 | 0.931 | 2564 | 199659 | 3512 | 11481 |  | 0.757 | 10545 | 153859 | 49312 | 3500 |
| RF-original | 0.937 | 1154 | 202373 | 798 | 12891 |  | 0.799 | 10223 | 163256 | 39915 | 3822 |
| RF-down | 0.768 | 10802 | 155974 | 47198 | 3242 |  | 0.164 | 13978 | 21709 | 181462 | 67 |
| RF-up | 0.917 | 4316 | 194969 | 8202 | 9729 |  | 0.543 | 13165 | 104712 | 98460 | 880 |
| RF-ROSE | 0.732 | 8966 | 150038 | 53134 | 5079 |  | 0.417 | 13476 | 77124 | 126047 | 569 |
| RF-SMOTE | 0.920 | 4210 | 195639 | 7532 | 9834 |  | 0.646 | 12417 | 127932 | 75240 | 1628 |
| RF-CC-Platt | 0.934 | 2468 | 200446 | 2725 | 11577 |  | 0.840 | 8880 | 173653 | 29519 | 5165 |
| RF-CC-IsoReg | 0.933 | 1946 | 200801 | 2371 | 12099 |  | 0.773 | 9757 | 158063 | 45108 | 4288 |
| RF-CC-Platt w/ SMOTE | 0.932 | 1669 | 200818 | 2353 | 12376 |  | 0.812 | 9516 | 166814 | 36358 | 4529 |
| RF-CC-IsoReg w/ SMOTE | 0.932 | 1331 | 201071 | 2100 | 12714 |  | 0.747 | 10594 | 151746 | 51425 | 3450 |
| RF-CSL-weight0.1 | 0.937 | 1058 | 202494 | 677 | 12987 |  | 0.800 | 10154 | 163726 | 39445 | 3890 |
| RF-CSL-weight0.01 | 0.937 | 1121 | 202387 | 784 | 12924 |  | 0.798 | 10191 | 163194 | 39978 | 3853 |
| Boost-original | 0.937 | 2468 | 200972 | 2200 | 11577 |  | 0.806 | 10418 | 164647 | 38524 | 3626 |
| Boost-RS-down | 0.766 | 10618 | 155684 | 47488 | 3427 |  | 0.173 | 13971 | 23589 | 179583 | 74 |
| Boost-RS-up | 0.812 | 10222 | 166151 | 37021 | 3823 |  | 0.139 | 14000 | 16275 | 186896 | 45 |
| Boost-RS-ROSE | 0.743 | 8238 | 153180 | 49991 | 5807 |  | 0.099 | 13954 | 7647 | 195524 | 91 |
| Boost-RS-SMOTE | 0.865 | 8020 | 179854 | 23318 | 6025 |  | 0.302 | 13745 | 51947 | 151224 | 299 |
| Boost-CC-Platt | 0.932 | 1964 | 200488 | 2684 | 12081 |  | 0.835 | 8376 | 172893 | 30279 | 5669 |
| Boost-CC-IsoReg | 0.935 | 592 | 202488 | 684 | 13452 |  | 0.832 | 8118 | 172608 | 30563 | 5926 |
| Boost-CC-Platt w/ SMOTE | 0.931 | 1261 | 200930 | 2242 | 12784 |  | 0.810 | 8369 | 167597 | 35574 | 5676 |
| Boost-CC-IsoReg w/ SMOTE | 0.927 | 1653 | 199692 | 3479 | 12391 |  | 0.788 | 8849 | 162303 | 40868 | 5195 |
| Boost-CSL-weight0.1 | 0.936 | 276 | 203063 | 108 | 13768 |  | 0.936 | 2604 | 200663 | 2508 | 11441 |
| Boost-CSL-weight0.01 | 0.936 | 65 | 203153 | 18 | 13980 |  | 0.937 | 1009 | 202546 | 625 | 13036 |

**Table S18.** Summary (median) of performance measures when data-generation model is boosting (Bold values indicate the best performing strategy in each ML model for each performance metric).

| **Analysis Model** | **MAPE** | **Brier Score** | **C-Statistic** | **Calibration in the large** | **Calibration slope** |
| --- | --- | --- | --- | --- | --- |
| LR-ME | 0.024 | 0.051 | 0.793 | -0.0007 | 0.901 |
| Bagg-original | 0.052 | 0.057 | 0.720 | -0.0100 | 0.601 |
| Bagg-down | 0.323 | 0.201 | **0.756** | -0.3221 | 0.552 |
| Bagg-up | 0.075 | 0.069 | 0.721 | -0.0294 | 0.156 |
| Bagg-ROSE | 0.182 | 0.167 | 0.749 | -0.1633 | 0.225 |
| Bagg-SMOTE | 0.080 | 0.070 | 0.731 | -0.0431 | 0.178 |
| Bagg-CC-Platt | **0.044** | **0.056** | 0.749 | 0.0036 | 1.297 |
| Bagg-CC-IsoReg | **0.043** | **0.056** | 0.713 | 0.0210 | **0.852** |
| Bagg-CC-Platt w/ SMOTE | 0.047 | 0.057 | 0.736 | 0.0028 | 2.358 |
| Bagg-CC-IsoReg w/ SMOTE | 0.054 | 0.064 | 0.692 | **-0.0016** | 0.665 |
| Bagg-CSL-weight0.1 | 0.052 | 0.057 | 0.723 | -0.0097 | 0.607 |
| Bagg-CSL-weight0.01 | 0.052 | 0.057 | 0.722 | -0.0099 | 0.605 |
| RF-original | **0.036** | **0.053** | 0.751 | 0.0114 | 0.793 |
| RF-down | 0.314 | 0.178 | 0.776 | -0.3143 | 0.937 |
| RF-up | 0.095 | 0.070 | 0.772 | -0.0856 | 0.617 |
| RF-ROSE | 0.191 | 0.140 | **0.783** | -0.1889 | 0.669 |
| RF-SMOTE | 0.071 | 0.064 | 0.774 | -0.0558 | 0.446 |
| RF-CC-Platt | 0.039 | 0.055 | 0.766 | 0.0028 | **1.056** |
| RF-CC-IsoReg | 0.044 | 0.058 | 0.717 | **-0.0007** | 0.874 |
| RF-CC-Platt w/ SMOTE | 0.038 | 0.055 | 0.774 | 0.0025 | 1.134 |
| RF-CC-IsoReg w/ SMOTE | 0.042 | 0.057 | 0.742 | 0.0012 | 0.887 |
| RF-CSL-weight0.1 | **0.036** | **0.053** | 0.752 | 0.0112 | 0.797 |
| RF-CSL-weight0.01 | **0.036** | **0.053** | 0.751 | 0.0115 | 0.794 |
| Boost-original | **0.024** | **0.051** | **0.796** | **-0.0002** | 0.903 |
| Boost-RS-down | 0.320 | 0.187 | 0.770 | -0.3197 | 0.832 |
| Boost-RS-up | 0.288 | 0.156 | 0.792 | -0.2883 | **1.011** |
| Boost-RS-ROSE | 0.254 | 0.172 | 0.765 | -0.2534 | 0.930 |
| Boost-RS-SMOTE | 0.080 | 0.070 | 0.731 | -0.0431 | 0.178 |
| Boost-CC-Platt | 0.044 | 0.056 | 0.747 | 0.0035 | 1.410 |
| Boost-CC-IsoReg | 0.043 | 0.056 | 0.711 | 0.0214 | 0.840 |
| Boost-CC-Platt w/ SMOTE | 0.047 | 0.057 | 0.736 | 0.0020 | 2.039 |
| Boost-CC-IsoReg w/ SMOTE | 0.054 | 0.064 | 0.694 | -0.0006 | 0.675 |
| Boost-CSL-weight0.1 | 0.055 | 0.059 | 0.792 | 0.0552 | 0.862 |
| Boost-CSL-weight0.01 | 0.061 | 0.061 | 0.781 | 0.0604 | 0.837 |

**Table S19.** Summary (mean) of confusion matrix when data-generation model is boosting.

|  | **Classification Threshold=0.50** | | | | |  | **Classification Threshold=0.063** | | | | |
| --- | --- | --- | --- | --- | --- | --- | --- | --- | --- | --- | --- |
| **Analysis Model** | **Accuracy** | **TP** | **TN** | **FP** | **FN** |  | **Accuracy** | **TP** | **TN** | **FP** | **FN** |
| LR-ME | 0.940 | 1602 | 202526 | 1073 | 12016 |  | 0.758 | 9384 | 155174 | 48424 | 4234 |
| Bagg-original | 0.933 | 1739 | 200916 | 2682 | 11879 |  | 0.728 | 8846 | 149356 | 54242 | 4772 |
| Bagg-down | 0.702 | 9357 | 143047 | 60551 | 4261 |  | 0.157 | 13300 | 20902 | 182696 | 317 |
| Bagg-up | 0.917 | 2196 | 196886 | 6712 | 11422 |  | 0.698 | 8704 | 142841 | 60757 | 4914 |
| Bagg-ROSE | 0.806 | 6481 | 168603 | 34995 | 7136 |  | 0.605 | 10686 | 120634 | 82964 | 2932 |
| Bagg-SMOTE | 0.913 | 2972 | 195418 | 8180 | 10646 |  | 0.664 | 9347 | 134830 | 68768 | 4271 |
| Bagg-CC-Platt | 0.936 | 540 | 202719 | 880 | 13078 |  | 0.798 | 7035 | 166326 | 37272 | 6583 |
| Bagg-CC-IsoReg | 0.937 | 331 | 203237 | 362 | 13286 |  | 0.859 | 5855 | 180770 | 22828 | 7763 |
| Bagg-CC-Platt w/ SMOTE | 0.936 | 301 | 202953 | 645 | 13317 |  | 0.752 | 6695 | 156670 | 46928 | 6923 |
| Bagg-CC-IsoReg w/ SMOTE | 0.931 | 1179 | 201034 | 2564 | 12439 |  | 0.800 | 7024 | 166718 | 36880 | 6594 |
| Bagg-CSL-weight0.1 | 0.933 | 1742 | 200937 | 2661 | 11876 |  | 0.729 | 8840 | 149580 | 54018 | 4778 |
| Bagg-CSL-weight0.01 | 0.933 | 1766 | 200889 | 2709 | 11852 |  | 0.730 | 8865 | 149673 | 53925 | 4753 |
| RF-original | 0.938 | 631 | 203191 | 407 | 12986 |  | 0.785 | 8429 | 162110 | 41488 | 5189 |
| RF-down | 0.742 | 9289 | 151935 | 51664 | 4329 |  | 0.096 | 13521 | 7415 | 196184 | 97 |
| RF-up | 0.919 | 3050 | 196604 | 6995 | 10568 |  | 0.468 | 11881 | 89845 | 113753 | 1737 |
| RF-ROSE | 0.801 | 7107 | 166779 | 36819 | 6510 |  | 0.417 | 12177 | 78396 | 125203 | 1441 |
| RF-SMOTE | 0.923 | 3009 | 197536 | 6062 | 10609 |  | 0.589 | 10981 | 117025 | 86573 | 2637 |
| RF-CC-Platt | 0.936 | 1117 | 202165 | 1433 | 12501 |  | 0.824 | 7278 | 171743 | 31855 | 6340 |
| RF-CC-IsoReg | 0.936 | 1063 | 202287 | 1311 | 12555 |  | 0.759 | 8407 | 156508 | 47090 | 5211 |
| RF-CC-Platt w/ SMOTE | 0.935 | 678 | 202511 | 1088 | 12940 |  | 0.799 | 8030 | 165609 | 37990 | 5588 |
| RF-CC-IsoReg w/ SMOTE | 0.936 | 730 | 202608 | 990 | 12887 |  | 0.790 | 8223 | 163376 | 40222 | 5395 |
| RF-CSL-weight0.1 | 0.938 | 648 | 203180 | 419 | 12970 |  | 0.784 | 8438 | 161895 | 41704 | 5180 |
| RF-CSL-weight0.01 | 0.938 | 674 | 203169 | 429 | 12944 |  | 0.786 | 8421 | 162238 | 41360 | 5197 |
| Boost-original | 0.939 | 1774 | 202276 | 1322 | 11844 |  | 0.781 | 9015 | 160549 | 43049 | 4603 |
| Boost-RS-down | 0.733 | 9219 | 149895 | 53703 | 4399 |  | 0.084 | 13546 | 4592 | 199006 | 72 |
| Boost-RS-up | 0.788 | 8858 | 162319 | 41279 | 4759 |  | 0.064 | 13614 | 311 | 203288 | 4 |
| Boost-RS-ROSE | 0.801 | 7035 | 166893 | 36706 | 6583 |  | 0.074 | 13548 | 2467 | 201131 | 70 |
| Boost-RS-SMOTE | 0.857 | 6888 | 179299 | 24300 | 6730 |  | 0.147 | 13313 | 18543 | 185056 | 305 |
| Boost-CC-Platt | 0.936 | 532 | 202773 | 825 | 13086 |  | 0.804 | 7003 | 167549 | 36049 | 6615 |
| Boost-CC-IsoReg | 0.937 | 321 | 203248 | 351 | 13297 |  | 0.859 | 5782 | 180769 | 22829 | 7836 |
| Boost-CC-Platt w/ SMOTE | 0.935 | 409 | 202754 | 844 | 13209 |  | 0.756 | 6758 | 157421 | 46177 | 6860 |
| Boost-CC-IsoReg w/ SMOTE | 0.931 | 1137 | 201090 | 2508 | 12481 |  | 0.805 | 6887 | 167917 | 35681 | 6731 |
| Boost-CSL-weight0.1 | 0.938 | 137 | 203561 | 37 | 13481 |  | 0.938 | 2060 | 201717 | 1881 | 11557 |
| Boost-CSL-weight0.01 | 0.937 | 25 | 203593 | 6 | 13593 |  | 0.939 | 663 | 203271 | 328 | 12954 |

**RESULTS: Heart Failure Dataset**

**Table S20.** Summary (median) of performance measures when data-generation model is logistic regression with non-linear effects (Bold values indicate the best performing strategy in each ML model for each performance metric).

| **Analysis Model** | **MAPE** | **Brier Score** | **C-Statistic** | **Calibration in the large** | **Calibration slope** |
| --- | --- | --- | --- | --- | --- |
| LR-Non-linear Effects | 0.028 | 0.082 | 0.799 | 0.0017 | 0.868 |
| LR-Only Main Effects | 0.043 | 0.086 | 0.776 | 0.0014 | 0.944 |
| Bagg-original | 0.059 | **0.088** | 0.752 | -0.0106 | 0.728 |
| Bagg-down | 0.296 | 0.196 | 0.762 | -0.2941 | 0.813 |
| Bagg-up | 0.088 | 0.100 | 0.725 | -0.0431 | 0.613 |
| Bagg-ROSE | 0.161 | 0.128 | **0.772** | -0.1566 | 0.695 |
| Bagg-SMOTE | 0.087 | 0.097 | 0.742 | -0.0523 | 0.709 |
| Bagg-CC-Platt | **0.055** | **0.088** | 0.763 | **0.0014** | **1.021** |
| Bagg-CC-IsoReg | 0.062 | 0.090 | 0.724 | 0.0331 | 0.761 |
| Bagg-CC-Platt w/ SMOTE | 0.061 | 0.091 | 0.753 | 0.0028 | 1.096 |
| Bagg-CC-IsoReg w/ SMOTE | 0.068 | 0.094 | 0.722 | 0.0048 | 0.738 |
| Bagg-CSL-weight0.1 | 0.058 | **0.088** | 0.752 | -0.0107 | 0.728 |
| Bagg-CSL-weight0.01 | 0.058 | **0.088** | 0.753 | -0.0105 | 0.732 |
| RF-original | **0.050** | **0.086** | 0.766 | **-0.0013** | 0.902 |
| RF-down | 0.301 | 0.186 | 0.772 | -0.2993 | 1.174 |
| RF-up | 0.079 | 0.093 | 0.766 | -0.0564 | 0.871 |
| RF-ROSE | 0.187 | 0.128 | **0.779** | -0.1851 | 1.042 |
| RF-SMOTE | 0.088 | 0.094 | 0.758 | -0.0632 | 0.979 |
| RF-CC-Platt | 0.051 | 0.087 | 0.769 | 0.0024 | 1.044 |
| RF-CC-IsoReg | 0.054 | 0.088 | 0.753 | 0.0018 | 0.931 |
| RF-CC-Platt w/ SMOTE | 0.058 | 0.090 | 0.759 | 0.0026 | **1.040** |
| RF-CC-IsoReg w/ SMOTE | 0.061 | 0.091 | 0.743 | 0.0027 | 0.913 |
| RF-CSL-weight0.1 | **0.049** | **0.086** | 0.765 | 0.0062 | 0.949 |
| RF-CSL-weight0.01 | **0.049** | **0.086** | 0.766 | 0.0067 | 0.946 |
| Boost-original | **0.036** | **0.083** | 0.791 | **0.0021** | 1.032 |
| Boost-RS-down | 0.288 | 0.181 | **0.792** | -0.2873 | 1.058 |
| Boost-RS-up | 0.283 | 0.173 | **0.792** | -0.2818 | 1.210 |
| Boost-RS-ROSE | 0.260 | 0.156 | 0.787 | -0.2595 | 1.331 |
| Boost-RS-SMOTE | 0.216 | 0.135 | 0.738 | -0.2034 | 1.477 |
| Boost-CC-Platt | 0.055 | 0.087 | 0.763 | 0.0022 | 1.028 |
| Boost-CC-IsoReg | 0.062 | 0.090 | 0.724 | 0.0329 | 0.762 |
| Boost-CC-Platt w/ SMOTE | 0.061 | 0.091 | 0.754 | 0.0032 | 1.081 |
| Boost-CC-IsoReg w/ SMOTE | 0.068 | 0.093 | 0.722 | 0.0058 | 0.739 |
| Boost-CSL-weight0.1 | 0.091 | 0.100 | 0.771 | 0.0904 | **0.995** |
| Boost-CSL-weight0.01 | 0.106 | 0.108 | 0.747 | 0.1062 | 0.988 |

**Table S21.** Summary (mean) of confusion matrix when data-generation model is logistic regression with non-linear effects.

|  | **Classification Threshold=0.50** | | | | |  | **Classification Threshold=0.11** | | | | |
| --- | --- | --- | --- | --- | --- | --- | --- | --- | --- | --- | --- |
| **Analysis Model** | **Accuracy** | **TP** | **TN** | **FP** | **FN** |  | **Accuracy** | **TP** | **TN** | **FP** | **FN** |
| LR-Non-linear Effects | 0.897 | 1272 | 47216 | 751 | 4842 |  | 0.753 | 4196 | 36511 | 11456 | 1918 |
| LR-Only Main Effects | 0.892 | 752 | 47468 | 500 | 5362 |  | 0.716 | 4224 | 34497 | 13470 | 1890 |
| Bagg-original | 0.889 | 1178 | 46924 | 1043 | 4936 |  | 0.701 | 4235 | 33656 | 14312 | 1879 |
| Bagg-down | 0.709 | 4177 | 34186 | 13781 | 1937 |  | 0.211 | 5999 | 5386 | 42581 | 114 |
| Bagg-up | 0.876 | 1328 | 46032 | 1935 | 4786 |  | 0.620 | 4555 | 28963 | 19004 | 1558 |
| Bagg-ROSE | 0.826 | 3150 | 41501 | 6467 | 2964 |  | 0.441 | 5494 | 18344 | 29623 | 620 |
| Bagg-SMOTE | 0.879 | 1277 | 46252 | 1715 | 4837 |  | 0.583 | 4845 | 26686 | 21281 | 1269 |
| Bagg-CC-Platt | 0.890 | 843 | 47312 | 655 | 5271 |  | 0.776 | 3588 | 38355 | 9612 | 2526 |
| Bagg-CC-IsoReg | 0.889 | 637 | 47464 | 503 | 5476 |  | 0.801 | 3314 | 40003 | 7964 | 2800 |
| Bagg-CC-Platt w/ SMOTE | 0.886 | 388 | 47527 | 440 | 5726 |  | 0.734 | 3838 | 35841 | 12126 | 2276 |
| Bagg-CC-IsoReg w/ SMOTE | 0.884 | 639 | 47156 | 811 | 5475 |  | 0.713 | 4018 | 34547 | 13421 | 2096 |
| Bagg-CSL-weight0.1 | 0.890 | 1181 | 46928 | 1039 | 4933 |  | 0.700 | 4242 | 33593 | 14374 | 1872 |
| Bagg-CSL-weight0.01 | 0.890 | 1187 | 46936 | 1031 | 4927 |  | 0.701 | 4240 | 33677 | 14290 | 1874 |
| RF-original | 0.892 | 783 | 47435 | 533 | 5331 |  | 0.713 | 4237 | 34297 | 13670 | 1877 |
| RF-down | 0.737 | 4029 | 35815 | 12152 | 2085 |  | 0.139 | 6091 | 1443 | 46524 | 23 |
| RF-up | 0.888 | 1068 | 46943 | 1025 | 5046 |  | 0.539 | 5134 | 24031 | 23936 | 980 |
| RF-ROSE | 0.837 | 3006 | 42256 | 5711 | 3108 |  | 0.281 | 5884 | 9337 | 38630 | 229 |
| RF-SMOTE | 0.885 | 931 | 46925 | 1043 | 5183 |  | 0.506 | 5218 | 22173 | 25794 | 895 |
| RF-CC-Platt | 0.892 | 873 | 47354 | 614 | 5241 |  | 0.773 | 3697 | 38118 | 9850 | 2417 |
| RF-CC-IsoReg | 0.891 | 759 | 47451 | 516 | 5355 |  | 0.742 | 3941 | 36201 | 11766 | 2173 |
| RF-CC-Platt w/ SMOTE | 0.887 | 509 | 47448 | 519 | 5605 |  | 0.736 | 3884 | 35902 | 12066 | 2230 |
| RF-CC-IsoReg w/ SMOTE | 0.886 | 435 | 47500 | 467 | 5679 |  | 0.697 | 4184 | 33517 | 14451 | 1929 |
| RF-CSL-weight0.1 | 0.891 | 578 | 47614 | 354 | 5536 |  | 0.729 | 4098 | 35311 | 12656 | 2015 |
| RF-CSL-weight0.01 | 0.891 | 570 | 47611 | 356 | 5544 |  | 0.731 | 4092 | 35431 | 12536 | 2022 |
| Boost-original | 0.896 | 1025 | 47439 | 528 | 5089 |  | 0.759 | 4056 | 36982 | 10986 | 2058 |
| Boost-RS-down | 0.737 | 4253 | 35606 | 12361 | 1861 |  | 0.163 | 6078 | 2733 | 45234 | 36 |
| Boost-RS-up | 0.758 | 4072 | 36935 | 11033 | 2042 |  | 0.133 | 6101 | 1110 | 46858 | 13 |
| Boost-RS-ROSE | 0.811 | 3434 | 40416 | 7551 | 2680 |  | 0.118 | 6111 | 283 | 47685 | 3 |
| Boost-RS-SMOTE | 0.846 | 1915 | 43851 | 4116 | 4199 |  | 0.124 | 6100 | 608 | 47360 | 14 |
| Boost-CC-Platt | 0.891 | 839 | 47334 | 633 | 5275 |  | 0.774 | 3616 | 38221 | 9746 | 2498 |
| Boost-CC-IsoReg | 0.889 | 637 | 47467 | 500 | 5477 |  | 0.801 | 3323 | 40000 | 7968 | 2791 |
| Boost-CC-Platt w/ SMOTE | 0.886 | 374 | 47535 | 432 | 5740 |  | 0.738 | 3807 | 36081 | 11886 | 2307 |
| Boost-CC-IsoReg w/ SMOTE | 0.884 | 640 | 47160 | 808 | 5474 |  | 0.716 | 3997 | 34738 | 13229 | 2117 |
| Boost-CSL-weight0.1 | 0.890 | 224 | 47909 | 58 | 5890 |  | 0.894 | 923 | 47428 | 540 | 5191 |
| Boost-CSL-weight0.01 | 0.888 | 50 | 47955 | 12 | 6064 |  | 0.891 | 336 | 47851 | 116 | 5777 |

**Table S22.** Summary (median) of performance measures when data-generation model is bagging (Bold values indicate the best performing strategy in each ML model for each performance metric).

| **Analysis Model** | **MAPE** | **Brier Score** | **C-Statistic** | **Calibration in the large** | **Calibration slope** |
| --- | --- | --- | --- | --- | --- |
| LR-Non-linear Effects | 0.106 | 0.086 | 0.798 | -0.0004 | 0.871 |
| LR-Only Main Effects | 0.118 | 0.089 | 0.766 | 0.0000 | 0.941 |
| Bagg-original | 0.115 | **0.088** | 0.767 | -0.0109 | 0.771 |
| Bagg-down | 0.314 | 0.192 | 0.772 | -0.2822 | 0.807 |
| Bagg-up | 0.140 | 0.098 | 0.740 | -0.0439 | 0.659 |
| Bagg-ROSE | 0.210 | 0.131 | 0.776 | -0.1576 | 0.698 |
| Bagg-SMOTE | 0.146 | 0.097 | 0.757 | -0.0533 | 0.768 |
| Bagg-CC-Platt | 0.112 | 0.088 | **0.780** | 0.0011 | 1.052 |
| Bagg-CC-IsoReg | **0.098** | 0.091 | 0.730 | 0.0298 | 0.737 |
| Bagg-CC-Platt w/ SMOTE | 0.120 | 0.091 | 0.767 | **0.0003** | 1.076 |
| Bagg-CC-IsoReg w/ SMOTE | 0.117 | 0.095 | 0.724 | 0.0009 | 0.716 |
| Bagg-CSL-weight0.1 | 0.114 | **0.088** | 0.768 | -0.0104 | 0.771 |
| Bagg-CSL-weight0.01 | 0.114 | **0.088** | 0.767 | -0.0107 | 0.768 |
| RF-original | 0.111 | **0.086** | 0.783 | -0.0032 | 0.961 |
| RF-down | 0.326 | 0.183 | 0.781 | -0.2915 | 1.184 |
| RF-up | 0.148 | 0.092 | 0.781 | -0.0559 | 0.982 |
| RF-ROSE | 0.240 | 0.131 | 0.784 | -0.1868 | 1.064 |
| RF-SMOTE | 0.155 | 0.094 | 0.773 | -0.0624 | 1.057 |
| RF-CC-Platt | 0.110 | 0.088 | **0.786** | **0.0011** | 1.063 |
| RF-CC-IsoReg | 0.110 | 0.088 | 0.767 | 0.0020 | 0.935 |
| RF-CC-Platt w/ SMOTE | 0.117 | 0.090 | 0.772 | 0.0019 | 1.056 |
| RF-CC-IsoReg w/ SMOTE | 0.118 | 0.092 | 0.750 | **0.0011** | 0.927 |
| RF-CSL-weight0.1 | **0.109** | **0.086** | 0.782 | 0.0039 | 1.014 |
| RF-CSL-weight0.01 | 0.110 | **0.086** | 0.782 | 0.0039 | 1.020 |
| Boost-original | 0.109 | **0.086** | **0.792** | 0.0006 | 1.031 |
| Boost-RS-down | 0.314 | 0.180 | 0.791 | -0.2833 | 1.049 |
| Boost-RS-up | 0.314 | 0.174 | 0.790 | -0.2806 | 1.202 |
| Boost-RS-ROSE | 0.299 | 0.156 | 0.785 | -0.2557 | 1.301 |
| Boost-RS-SMOTE | 0.274 | 0.138 | 0.729 | -0.2008 | 1.401 |
| Boost-CC-Platt | 0.112 | 0.088 | 0.781 | 0.0011 | 1.050 |
| Boost-CC-IsoReg | **0.099** | 0.091 | 0.730 | 0.0299 | 0.738 |
| Boost-CC-Platt w/ SMOTE | 0.120 | 0.091 | 0.767 | 0.0009 | 1.077 |
| Boost-CC-IsoReg w/ SMOTE | 0.118 | 0.096 | 0.725 | **-0.0001** | 0.716 |
| Boost-CSL-weight0.1 | 0.100 | 0.104 | 0.773 | 0.0922 | **0.989** |
| Boost-CSL-weight0.01 | 0.111 | 0.112 | 0.753 | 0.1092 | 0.930 |

**Table S23.** Summary (mean) of confusion matrix when data-generation model is bagging.

|  | **Classification Threshold=0.50** | | | | |  | **Classification Threshold=0.11** | | | | |
| --- | --- | --- | --- | --- | --- | --- | --- | --- | --- | --- | --- |
| **Analysis Model** | **Accuracy** | **TP** | **TN** | **FP** | **FN** |  | **Accuracy** | **TP** | **TN** | **FP** | **FN** |
| LR-Non-linear Effects | 0.891 | 1269 | 46918 | 831 | 5063 |  | 0.736 | 4484 | 35329 | 12420 | 1848 |
| LR-Only Main Effects | 0.888 | 781 | 47258 | 491 | 5551 |  | 0.695 | 4433 | 33162 | 14587 | 1899 |
| Bagg-original | 0.889 | 1446 | 46643 | 1107 | 4886 |  | 0.701 | 4591 | 33317 | 14432 | 1741 |
| Bagg-down | 0.710 | 4409 | 33973 | 13776 | 1923 |  | 0.254 | 6184 | 7578 | 40171 | 148 |
| Bagg-up | 0.876 | 1583 | 45768 | 1982 | 4749 |  | 0.620 | 4929 | 28611 | 19138 | 1403 |
| Bagg-ROSE | 0.821 | 3301 | 41112 | 6637 | 3031 |  | 0.437 | 5778 | 17858 | 29891 | 554 |
| Bagg-SMOTE | 0.878 | 1563 | 45917 | 1832 | 4769 |  | 0.584 | 5176 | 26393 | 21356 | 1156 |
| Bagg-CC-Platt | 0.889 | 1021 | 47031 | 718 | 5311 |  | 0.774 | 3927 | 37920 | 9829 | 2405 |
| Bagg-CC-IsoReg | 0.887 | 804 | 47184 | 566 | 5528 |  | 0.812 | 3443 | 40492 | 7258 | 2889 |
| Bagg-CC-Platt w/ SMOTE | 0.884 | 604 | 47223 | 526 | 5728 |  | 0.728 | 4131 | 35265 | 12484 | 2201 |
| Bagg-CC-IsoReg w/ SMOTE | 0.882 | 821 | 46858 | 891 | 5511 |  | 0.734 | 4045 | 35675 | 12074 | 2287 |
| Bagg-CSL-weight0.1 | 0.889 | 1450 | 46644 | 1106 | 4882 |  | 0.703 | 4583 | 33421 | 14328 | 1749 |
| Bagg-CSL-weight0.01 | 0.889 | 1459 | 46634 | 1116 | 4873 |  | 0.701 | 4598 | 33336 | 14413 | 1734 |
| RF-original | 0.891 | 1001 | 47185 | 564 | 5331 |  | 0.705 | 4636 | 33468 | 14282 | 1696 |
| RF-down | 0.740 | 4237 | 35781 | 11968 | 2095 |  | 0.155 | 6310 | 2091 | 45658 | 22 |
| RF-up | 0.889 | 1285 | 46768 | 981 | 5047 |  | 0.537 | 5451 | 23605 | 24145 | 881 |
| RF-ROSE | 0.830 | 3128 | 41755 | 5994 | 3204 |  | 0.275 | 6165 | 8696 | 39053 | 167 |
| RF-SMOTE | 0.885 | 1201 | 46668 | 1081 | 5131 |  | 0.508 | 5534 | 21916 | 25833 | 798 |
| RF-CC-Platt | 0.890 | 1104 | 47014 | 735 | 5228 |  | 0.771 | 4010 | 37672 | 10077 | 2322 |
| RF-CC-IsoReg | 0.888 | 964 | 47084 | 665 | 5368 |  | 0.758 | 4073 | 36911 | 10838 | 2259 |
| RF-CC-Platt w/ SMOTE | 0.885 | 692 | 47181 | 568 | 5640 |  | 0.732 | 4172 | 35410 | 12339 | 2160 |
| RF-CC-IsoReg w/ SMOTE | 0.884 | 540 | 47291 | 459 | 5792 |  | 0.723 | 4199 | 34895 | 12854 | 2133 |
| RF-CSL-weight0.1 | 0.890 | 766 | 47368 | 382 | 5566 |  | 0.716 | 4508 | 34238 | 13511 | 1824 |
| RF-CSL-weight0.01 | 0.890 | 755 | 47384 | 365 | 5577 |  | 0.716 | 4514 | 34218 | 13531 | 1818 |
| Boost-original | 0.891 | 1052 | 47153 | 596 | 5280 |  | 0.744 | 4294 | 35918 | 11832 | 2038 |
| Boost-RS-down | 0.740 | 4353 | 35675 | 12074 | 1979 |  | 0.155 | 6309 | 2085 | 45664 | 23 |
| Boost-RS-up | 0.755 | 4185 | 36643 | 11106 | 2147 |  | 0.128 | 6327 | 589 | 47160 | 5 |
| Boost-RS-ROSE | 0.809 | 3455 | 40289 | 7461 | 2877 |  | 0.118 | 6332 | 71 | 47678 | 0 |
| Boost-RS-SMOTE | 0.840 | 2014 | 43417 | 4333 | 4318 |  | 0.122 | 6324 | 298 | 47451 | 8 |
| Boost-CC-Platt | 0.888 | 1032 | 47017 | 732 | 5300 |  | 0.774 | 3913 | 37950 | 9799 | 2419 |
| Boost-CC-IsoReg | 0.887 | 795 | 47187 | 563 | 5537 |  | 0.812 | 3443 | 40467 | 7282 | 2889 |
| Boost-CC-Platt w/ SMOTE | 0.884 | 549 | 47266 | 484 | 5783 |  | 0.727 | 4148 | 35165 | 12584 | 2184 |
| Boost-CC-IsoReg w/ SMOTE | 0.881 | 809 | 46863 | 886 | 5523 |  | 0.733 | 4084 | 35541 | 12208 | 2248 |
| Boost-CSL-weight0.1 | 0.885 | 186 | 47681 | 68 | 6146 |  | 0.890 | 1082 | 47041 | 708 | 5250 |
| Boost-CSL-weight0.01 | 0.883 | 47 | 47729 | 20 | 6285 |  | 0.887 | 382 | 47579 | 170 | 5950 |

**Table S24.** Summary (median) of performance measures when data-generation model is random forest (Bold values indicate the best performing strategy in each ML model for each performance metric).

| **Analysis Model** | **MAPE** | **Brier Score** | **C-Statistic** | **Calibration in the large** | **Calibration slope** |
| --- | --- | --- | --- | --- | --- |
| LR-Non-linear Effects | 0.095 | 0.084 | 0.792 | 0.0013 | 0.855 |
| LR-Only Main Effects | 0.106 | 0.087 | 0.765 | 0.0013 | 0.926 |
| Bagg-original | 0.105 | **0.087** | 0.757 | -0.0104 | 0.730 |
| Bagg-down | 0.313 | 0.194 | 0.765 | -0.2886 | 0.797 |
| Bagg-up | 0.130 | 0.097 | 0.732 | -0.0421 | 0.637 |
| Bagg-ROSE | 0.194 | 0.127 | 0.770 | -0.1486 | 0.661 |
| Bagg-SMOTE | 0.134 | 0.095 | 0.749 | -0.0496 | 0.734 |
| Bagg-CC-Platt | 0.098 | **0.087** | **0.772** | **0.0055** | **0.961** |
| Bagg-CC-IsoReg | **0.091** | 0.090 | 0.724 | 0.0338 | 0.689 |
| Bagg-CC-Platt w/ SMOTE | 0.104 | 0.089 | 0.760 | 0.0066 | 0.959 |
| Bagg-CC-IsoReg w/ SMOTE | 0.106 | 0.094 | 0.717 | 0.0065 | 0.658 |
| Bagg-CSL-weight0.1 | 0.105 | **0.087** | 0.757 | -0.0102 | 0.732 |
| Bagg-CSL-weight0.01 | 0.105 | **0.087** | 0.757 | -0.0105 | 0.729 |
| RF-original | 0.102 | **0.084** | 0.775 | **-0.0017** | 0.919 |
| RF-down | 0.320 | 0.184 | 0.776 | -0.2949 | 1.184 |
| RF-up | 0.136 | 0.090 | 0.774 | -0.0536 | 0.914 |
| RF-ROSE | 0.214 | 0.123 | **0.779** | -0.1684 | 0.988 |
| RF-SMOTE | 0.144 | 0.092 | 0.766 | -0.0619 | **1.010** |
| RF-CC-Platt | 0.095 | 0.086 | **0.779** | 0.0051 | 0.944 |
| RF-CC-IsoReg | 0.099 | 0.088 | 0.754 | 0.0045 | 0.810 |
| RF-CC-Platt w/ SMOTE | 0.103 | 0.088 | 0.765 | 0.0066 | 1.016 |
| RF-CC-IsoReg w/ SMOTE | 0.106 | 0.090 | 0.740 | 0.0053 | 0.836 |
| RF-CSL-weight0.1 | **0.097** | **0.084** | 0.773 | 0.0064 | 0.959 |
| RF-CSL-weight0.01 | 0.098 | **0.084** | 0.773 | 0.0064 | 0.962 |
| Boost-original | 0.097 | **0.084** | **0.788** | **0.0019** | **1.003** |
| Boost-RS-down | 0.310 | 0.184 | 0.784 | -0.2886 | 0.981 |
| Boost-RS-up | 0.307 | 0.175 | 0.786 | -0.2832 | 1.155 |
| Boost-RS-ROSE | 0.287 | 0.154 | 0.783 | -0.2546 | 1.240 |
| Boost-RS-SMOTE | 0.262 | 0.136 | 0.732 | -0.2022 | 1.389 |
| Boost-CC-Platt | 0.098 | 0.087 | 0.773 | 0.0063 | 0.968 |
| Boost-CC-IsoReg | **0.091** | 0.090 | 0.722 | 0.0336 | 0.681 |
| Boost-CC-Platt w/ SMOTE | 0.104 | 0.090 | 0.760 | 0.0071 | 0.974 |
| Boost-CC-IsoReg w/ SMOTE | 0.106 | 0.094 | 0.716 | 0.0070 | 0.658 |
| Boost-CSL-weight0.1 | 0.096 | 0.101 | 0.771 | 0.0912 | 0.995 |
| Boost-CSL-weight0.01 | 0.107 | 0.108 | 0.743 | 0.1061 | 0.964 |

**Table S25.** Summary (mean) of confusion matrix when data-generation model is random forest.

|  | **Threshold=0.50** | | | | |  | **Threshold=0.11** | | | | |
| --- | --- | --- | --- | --- | --- | --- | --- | --- | --- | --- | --- |
| **Model** | **Accuracy** | **TP** | **TN** | **FP** | **FN** |  | **Accuracy** | **TP** | **TN** | **FP** | **FN** |
| LR-Non-linear Effects | 0.894 | 1084 | 47266 | 732 | 4999 |  | 0.744 | 4157 | 36076 | 11922 | 1926 |
| LR-Only Main Effects | 0.892 | 700 | 47518 | 480 | 5384 |  | 0.714 | 4101 | 34491 | 13507 | 1983 |
| Bagg-original | 0.890 | 1310 | 46835 | 1162 | 4774 |  | 0.709 | 4263 | 34090 | 13907 | 1821 |
| Bagg-down | 0.705 | 4196 | 33930 | 14067 | 1888 |  | 0.243 | 5947 | 7170 | 40827 | 137 |
| Bagg-up | 0.880 | 1443 | 46133 | 1864 | 4641 |  | 0.627 | 4573 | 29349 | 18648 | 1511 |
| Bagg-ROSE | 0.825 | 3082 | 41526 | 6471 | 3002 |  | 0.470 | 5414 | 20023 | 27975 | 670 |
| Bagg-SMOTE | 0.882 | 1380 | 46314 | 1683 | 4704 |  | 0.598 | 4834 | 27502 | 20496 | 1250 |
| Bagg-CC-Platt | 0.890 | 979 | 47175 | 822 | 5105 |  | 0.795 | 3457 | 39546 | 8452 | 2627 |
| Bagg-CC-IsoReg | 0.890 | 834 | 47293 | 704 | 5249 |  | 0.805 | 3313 | 40225 | 7772 | 2771 |
| Bagg-CC-Platt w/ SMOTE | 0.888 | 583 | 47436 | 561 | 5501 |  | 0.758 | 3655 | 37317 | 10680 | 2429 |
| Bagg-CC-IsoReg w/ SMOTE | 0.884 | 864 | 46927 | 1070 | 5219 |  | 0.721 | 3961 | 35020 | 12977 | 2123 |
| Bagg-CSL-weight0.1 | 0.891 | 1309 | 46859 | 1138 | 4774 |  | 0.708 | 4265 | 34018 | 13979 | 1819 |
| Bagg-CSL-weight0.01 | 0.890 | 1319 | 46836 | 1161 | 4765 |  | 0.708 | 4256 | 34011 | 13987 | 1827 |
| RF-original | 0.894 | 874 | 47453 | 545 | 5210 |  | 0.716 | 4297 | 34446 | 13551 | 1786 |
| RF-down | 0.740 | 4004 | 36006 | 11991 | 2080 |  | 0.152 | 6059 | 2185 | 45813 | 25 |
| RF-up | 0.891 | 1187 | 47004 | 993 | 4897 |  | 0.557 | 5097 | 25007 | 22991 | 987 |
| RF-ROSE | 0.840 | 2826 | 42614 | 5384 | 3258 |  | 0.324 | 5814 | 11712 | 36285 | 270 |
| RF-SMOTE | 0.889 | 1077 | 46985 | 1012 | 5007 |  | 0.518 | 5220 | 22809 | 25188 | 864 |
| RF-CC-Platt | 0.893 | 1062 | 47207 | 790 | 5022 |  | 0.798 | 3508 | 39627 | 8370 | 2576 |
| RF-CC-IsoReg | 0.892 | 743 | 47494 | 504 | 5341 |  | 0.725 | 4070 | 35122 | 12875 | 2014 |
| RF-CC-Platt w/ SMOTE | 0.889 | 583 | 47520 | 478 | 5501 |  | 0.756 | 3702 | 37192 | 10806 | 2382 |
| RF-CC-IsoReg w/ SMOTE | 0.888 | 627 | 47409 | 588 | 5457 |  | 0.699 | 4142 | 33643 | 14354 | 1942 |
| RF-CSL-weight0.1 | 0.893 | 676 | 47622 | 376 | 5408 |  | 0.734 | 4124 | 35579 | 12418 | 1959 |
| RF-CSL-weight0.01 | 0.893 | 659 | 47630 | 368 | 5425 |  | 0.733 | 4132 | 35534 | 12463 | 1951 |
| Boost-original | 0.895 | 972 | 47415 | 583 | 5112 |  | 0.757 | 3966 | 36950 | 11047 | 2118 |
| Boost-RS-down | 0.733 | 4143 | 35501 | 12496 | 1941 |  | 0.152 | 6062 | 2138 | 45860 | 22 |
| Boost-RS-up | 0.751 | 4004 | 36601 | 11396 | 2080 |  | 0.127 | 6079 | 800 | 47197 | 5 |
| Boost-RS-ROSE | 0.811 | 3272 | 40598 | 7400 | 2812 |  | 0.116 | 6083 | 168 | 47829 | 1 |
| Boost-RS-SMOTE | 0.843 | 1930 | 43637 | 4361 | 4154 |  | 0.118 | 6077 | 287 | 47711 | 7 |
| Boost-CC-Platt | 0.891 | 952 | 47213 | 785 | 5132 |  | 0.794 | 3463 | 39483 | 8514 | 2620 |
| Boost-CC-IsoReg | 0.890 | 839 | 47296 | 702 | 5245 |  | 0.805 | 3308 | 40217 | 7781 | 2776 |
| Boost-CC-Platt w/ SMOTE | 0.888 | 545 | 47471 | 527 | 5539 |  | 0.760 | 3621 | 37460 | 10537 | 2463 |
| Boost-CC-IsoReg w/ SMOTE | 0.884 | 862 | 46927 | 1070 | 5222 |  | 0.721 | 3948 | 35031 | 12966 | 2136 |
| Boost-CSL-weight0.1 | 0.889 | 119 | 47955 | 42 | 5965 |  | 0.893 | 905 | 47399 | 599 | 5179 |
| Boost-CSL-weight0.01 | 0.888 | 27 | 47989 | 8 | 6057 |  | 0.890 | 265 | 47877 | 121 | 5819 |

**Table S26.** Summary (median) of performance measures when data-generation model is boosting (Bold values indicate the best performing strategy in each ML model for each performance metric).

| **Analysis Model** | **MAPE** | **Brier Score** | **C-Statistic** | **Calibration in the large** | **Calibration slope** |
| --- | --- | --- | --- | --- | --- |
| LR-Only Main Effects | 0.039 | 0.091 | 0.719 | -0.0028 | 0.904 |
| LR-Non-linear Effects | 0.033 | 0.089 | 0.731 | -0.0025 | 0.831 |
| Bagg-original | 0.063 | 0.095 | 0.702 | -0.0185 | 0.614 |
| Bagg-down | 0.328 | 0.221 | 0.699 | -0.3267 | 0.659 |
| Bagg-up | 0.092 | 0.107 | 0.680 | -0.0537 | 0.529 |
| Bagg-ROSE | 0.179 | 0.142 | **0.717** | -0.1760 | 0.613 |
| Bagg-SMOTE | 0.088 | 0.103 | 0.692 | -0.0561 | 0.583 |
| Bagg-CC-Platt | **0.048** | **0.092** | 0.703 | -0.0046 | 0.956 |
| Bagg-CC-IsoReg | 0.055 | 0.094 | 0.685 | 0.0237 | 0.834 |
| Bagg-CC-Platt w/ SMOTE | 0.055 | 0.095 | 0.691 | -0.0053 | **1.002** |
| Bagg-CC-IsoReg w/ SMOTE | 0.063 | 0.097 | 0.675 | **-0.0005** | 0.743 |
| Bagg-CSL-weight0.1 | 0.063 | 0.095 | 0.700 | -0.0182 | 0.608 |
| Bagg-CSL-weight0.01 | 0.063 | 0.095 | 0.701 | -0.0182 | 0.608 |
| RF-original | 0.049 | 0.092 | 0.711 | -0.0072 | 0.751 |
| RF-down | 0.323 | 0.205 | 0.714 | -0.3231 | **1.011** |
| RF-up | 0.083 | 0.100 | 0.708 | -0.0677 | 0.704 |
| RF-ROSE | 0.219 | 0.149 | **0.723** | -0.2188 | 0.889 |
| RF-SMOTE | 0.087 | 0.100 | 0.701 | -0.0671 | 0.791 |
| RF-CC-Platt | **0.044** | 0.092 | 0.711 | -0.0047 | 0.986 |
| RF-CC-IsoReg | 0.048 | 0.093 | 0.699 | **-0.0044** | 0.890 |
| RF-CC-Platt w/ SMOTE | 0.052 | 0.094 | 0.698 | -0.0058 | 1.045 |
| RF-CC-IsoReg w/ SMOTE | 0.057 | 0.095 | 0.686 | -0.0055 | 0.885 |
| RF-CSL-weight0.1 | 0.046 | **0.091** | 0.711 | 0.0021 | 0.782 |
| RF-CSL-weight0.01 | 0.046 | **0.091** | 0.711 | 0.0024 | 0.783 |
| Boost-original | **0.025** | **0.088** | **0.740** | -0.0024 | 0.968 |
| Boost-RS-down | 0.317 | 0.203 | 0.731 | -0.3171 | 0.921 |
| Boost-RS-up | 0.307 | 0.192 | 0.737 | -0.3069 | 1.065 |
| Boost-RS-ROSE | 0.276 | 0.168 | 0.735 | -0.2759 | 1.198 |
| Boost-RS-SMOTE | 0.219 | 0.142 | 0.683 | -0.2115 | 1.338 |
| Boost-CC-Platt | 0.048 | 0.092 | 0.703 | -0.0043 | **0.993** |
| Boost-CC-IsoReg | 0.055 | 0.094 | 0.686 | 0.0231 | 0.834 |
| Boost-CC-Platt w/ SMOTE | 0.055 | 0.095 | 0.692 | -0.0049 | 1.010 |
| Boost-CC-IsoReg w/ SMOTE | 0.063 | 0.097 | 0.676 | **-0.0008** | 0.746 |
| Boost-CSL-weight0.1 | 0.091 | 0.102 | 0.735 | 0.0910 | 0.935 |
| Boost-CSL-weight0.01 | 0.106 | 0.109 | 0.728 | 0.1062 | 0.917 |

**Table S27.** Summary (mean) of confusion matrix when data-generation model is boosting.

|  | **Classification Threshold=0.50** | | | | |  | **Classification Threshold=0.11** | | | | |
| --- | --- | --- | --- | --- | --- | --- | --- | --- | --- | --- | --- |
| **Analysis Model** | **Accuracy** | **TP** | **TN** | **FP** | **FN** |  | **Accuracy** | **TP** | **TN** | **FP** | **FN** |
| LR-Only Main Effects | 0.888 | 400 | 47639 | 364 | 5678 |  | 0.674 | 3956 | 32483 | 15521 | 2122 |
| LR-Non-linear Effects | 0.891 | 765 | 47441 | 562 | 5313 |  | 0.712 | 3806 | 34677 | 13326 | 2272 |
| Bagg-original | 0.884 | 848 | 46975 | 1028 | 5230 |  | 0.655 | 3934 | 31485 | 16518 | 2144 |
| Bagg-down | 0.668 | 3818 | 32283 | 15720 | 2260 |  | 0.155 | 5969 | 2436 | 45567 | 109 |
| Bagg-up | 0.870 | 1036 | 46039 | 1964 | 5042 |  | 0.561 | 4308 | 26032 | 21971 | 1770 |
| Bagg-ROSE | 0.809 | 2765 | 40965 | 7038 | 3313 |  | 0.355 | 5384 | 13811 | 34192 | 694 |
| Bagg-SMOTE | 0.875 | 881 | 46452 | 1551 | 5197 |  | 0.538 | 4544 | 24562 | 23441 | 1534 |
| Bagg-CC-Platt | 0.887 | 450 | 47510 | 494 | 5628 |  | 0.721 | 3431 | 35550 | 12453 | 2647 |
| Bagg-CC-IsoReg | 0.887 | 168 | 47810 | 193 | 5910 |  | 0.772 | 2931 | 38796 | 9207 | 3147 |
| Bagg-CC-Platt w/ SMOTE | 0.886 | 152 | 47738 | 265 | 5926 |  | 0.676 | 3629 | 32948 | 15055 | 2449 |
| Bagg-CC-IsoReg w/ SMOTE | 0.885 | 176 | 47693 | 310 | 5902 |  | 0.687 | 3514 | 33632 | 14371 | 2564 |
| Bagg-CSL-weight0.1 | 0.884 | 852 | 46967 | 1036 | 5226 |  | 0.656 | 3911 | 31539 | 16464 | 2167 |
| Bagg-CSL-weight0.01 | 0.884 | 856 | 46957 | 1046 | 5222 |  | 0.656 | 3911 | 31567 | 16436 | 2167 |
| RF-original | 0.887 | 528 | 47463 | 540 | 5550 |  | 0.676 | 3863 | 32669 | 15334 | 2215 |
| RF-down | 0.711 | 3649 | 34809 | 13194 | 2429 |  | 0.119 | 6064 | 358 | 47645 | 14 |
| RF-up | 0.883 | 846 | 46927 | 1076 | 5232 |  | 0.465 | 4935 | 20206 | 27797 | 1143 |
| RF-ROSE | 0.810 | 2823 | 40983 | 7020 | 3255 |  | 0.182 | 5909 | 3952 | 44052 | 169 |
| RF-SMOTE | 0.881 | 646 | 47022 | 981 | 5432 |  | 0.462 | 4932 | 20054 | 27949 | 1146 |
| RF-CC-Platt | 0.888 | 494 | 47527 | 476 | 5584 |  | 0.725 | 3491 | 35716 | 12287 | 2587 |
| RF-CC-IsoReg | 0.887 | 508 | 47469 | 534 | 5570 |  | 0.758 | 3209 | 37785 | 10218 | 2869 |
| RF-CC-Platt w/ SMOTE | 0.886 | 188 | 47734 | 269 | 5890 |  | 0.669 | 3764 | 32403 | 15600 | 2314 |
| RF-CC-IsoReg w/ SMOTE | 0.887 | 109 | 47844 | 160 | 5969 |  | 0.658 | 3746 | 31858 | 16145 | 2332 |
| RF-CSL-weight0.1 | 0.888 | 372 | 47646 | 358 | 5706 |  | 0.701 | 3699 | 34229 | 13775 | 2379 |
| RF-CSL-weight0.01 | 0.888 | 375 | 47652 | 351 | 5703 |  | 0.702 | 3704 | 34246 | 13757 | 2374 |
| Boost-original | 0.892 | 731 | 47529 | 474 | 5347 |  | 0.738 | 3677 | 36223 | 11780 | 2401 |
| Boost-RS-down | 0.712 | 3799 | 34730 | 13274 | 2279 |  | 0.116 | 6070 | 212 | 47791 | 8 |
| Boost-RS-up | 0.739 | 3644 | 36326 | 11677 | 2434 |  | 0.113 | 6076 | 51 | 47952 | 2 |
| Boost-RS-ROSE | 0.803 | 3012 | 40407 | 7596 | 3066 |  | 0.112 | 6078 | 0 | 48003 | 0 |
| Boost-RS-SMOTE | 0.848 | 1311 | 44530 | 3473 | 4767 |  | 0.116 | 6067 | 190 | 47813 | 11 |
| Boost-CC-Platt | 0.887 | 393 | 47574 | 429 | 5685 |  | 0.717 | 3443 | 35352 | 12651 | 2635 |
| Boost-CC-IsoReg | 0.887 | 178 | 47806 | 198 | 5900 |  | 0.770 | 2950 | 38693 | 9310 | 3128 |
| Boost-CC-Platt w/ SMOTE | 0.885 | 155 | 47730 | 273 | 5923 |  | 0.674 | 3654 | 32808 | 15195 | 2424 |
| Boost-CC-IsoReg w/ SMOTE | 0.885 | 180 | 47678 | 325 | 5898 |  | 0.685 | 3538 | 33516 | 14487 | 2540 |
| Boost-CSL-weight0.1 | 0.889 | 68 | 47984 | 19 | 6010 |  | 0.891 | 788 | 47387 | 616 | 5290 |
| Boost-CSL-weight0.01 | 0.888 | 7 | 48001 | 2 | 6071 |  | 0.890 | 214 | 47910 | 93 | 5864 |

**4. Distribution of the linear predictor of DGMs**

**MINAP Dataset**

**
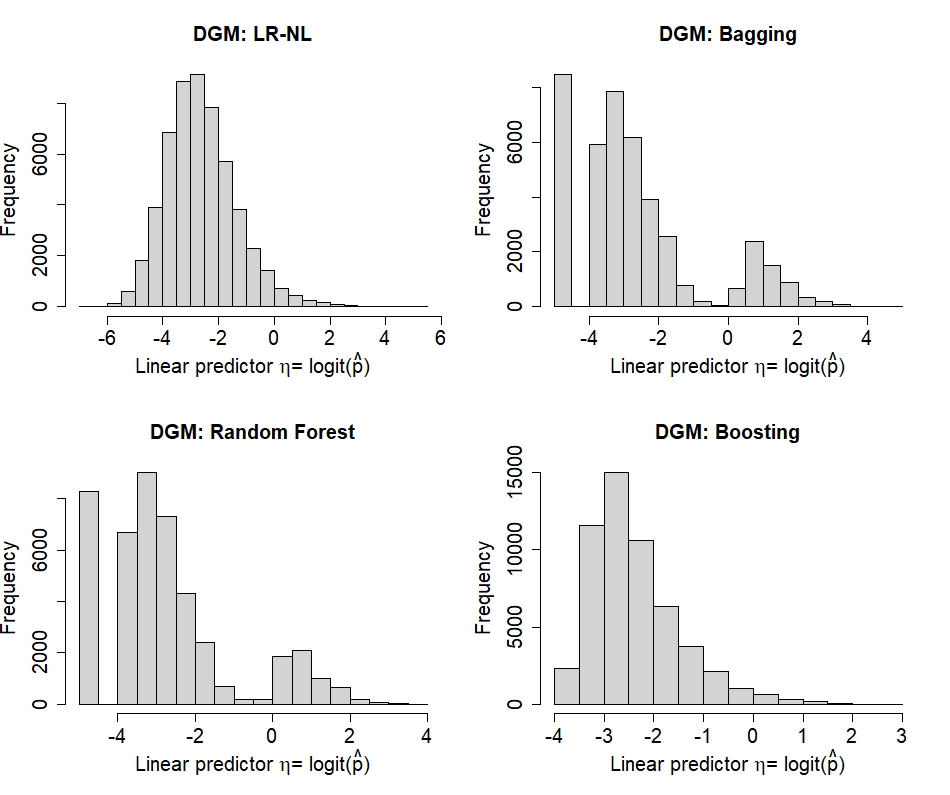
**

**Figure S1.** Distribution of linear predictor of DGMs for MINAP Dataset

**Heart Failure Dataset**

**
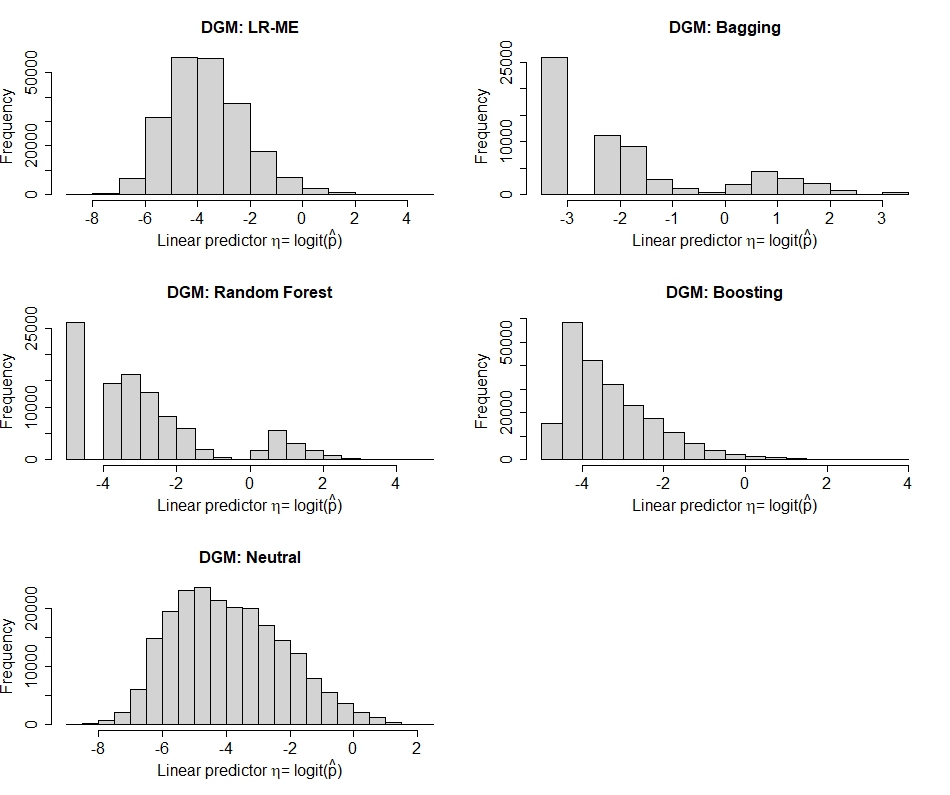
**

**Figure S2.** Distribution of linear predictor of DGMs for HF Dataset
